# Poor Prospects for Avian Biodiversity in Amazonian Oil Palm

**DOI:** 10.1371/journal.pone.0122432

**Published:** 2015-05-08

**Authors:** Alexander C. Lees, Nárgila G. Moura, Arlete Silva de Almeida, Ima C. G. Vieira

**Affiliations:** 1 Museu Paraense Emílio Goeldi, Belém, PA, Brazil; 2 Programa de Pós-Graduação em Ciências Ambientais, Universidade Federal do Pará/ Museu Paraense Emílio Goeldi, Belém—Pará, Brazil; Instituto de Pesquisas Ecológicas, BRAZIL

## Abstract

Expansion of oil palm plantations across the humid tropics has precipitated massive loss of tropical forest habitats and their associated speciose biotas. Oil palm plantation monocultures have been identified as an emerging threat to Amazonian biodiversity, but there are no quantitative studies exploring the impact of these plantations on the biome’s biota. Understanding these impacts is extremely important given the rapid projected expansion of oil palm cultivation in the basin. Here we investigate the biodiversity value of oil palm plantations in comparison with other dominant regional land-uses in Eastern Amazonia. We carried out bird surveys in oil palm plantations of varying ages, primary and secondary forests, and cattle pastures. We found that oil palm plantations retained impoverished avian communities with a similar species composition to pastures and agrarian land-uses and did not offer habitat for most forest-associated species, including restricted range species and species of conservation concern. On the other hand, the forests that the oil palm companies are legally obliged to protect hosted a relatively species-rich community including several globally-threatened bird species. We consider oil palm to be no less detrimental to regional biodiversity than other agricultural land-uses and that political pressure exerted by large landowners to allow oil palm to count as a substitute for native forest vegetation in private landholdings with forest restoration deficits would have dire consequences for regional biodiversity.

## Introduction

Globally the demand for food, animal feed, and fuel continues to increase at unprecedented rates, yet land available for agriculture is shrinking in many parts of the world [1], [2]. World food demand is forecast to more than double by 2050 [1], brought about both by a growing human population and even more rapid rises in meat consumption [3]. Together with the rapidly growing biofuel market [4]–[6], they represent one of the most pervasive threats to tropical biodiversity, driving conversion of natural ecosystems [1], [7], [8]. Oil palm (*Elaeis* spp.) plantations are now a dominant tropical land-use occupying over 16 million hectares [9]. It is estimated that 74% of global palm oil usage is for food products and 24% for industrial purposes, the latter predominantly for biodiesel [10]. Production is especially prevalent in Indo-Malaysia (80% of the global total), but plantation acreage is also increasing rapidly in the Afro- and Neotropics [11], [12]. This rapid expansion is likely to continue for decades given both high profitability and high demand. Proponents of palm oil emphasize that its main alternatives, including soy, sunflower, and canola oils, have production efficiencies just 10–20% as high as palm oil (on a per-hectare basis) and would therefore require much larger areas of cultivated land to have a similar benefit [13], moreover, the industry is also highly lucrative and could potentially create thousands of new jobs and raise regional standards of living.

Although widely flagged as a green fuel, from climate-change and biodiversity perspectives, such advantages are diminished should palm oil production contribute either directly or indirectly to deforestation [10], [11]. This has proven to be the case in many parts of the world, where expansion has come at the expense of both undisturbed and logged primary forest [8], [12], [14] despite the high biodiversity value associated with even degraded primary forests [15]. This loss is also partially due to plantation owners using timber revenues (from primary forests) to provide set-up costs for plantation establishment and maintenance [14]. Thus the question of how to make oil palm a more environmentally friendly crop becomes of critical conservation importance [16–18]. As mitigation measures, *in situ* practices to enhance local biodiversity; such as production of oil palm beneath shade trees, diverse agro-forestry on plantation boundaries, and maintenance of forest patches within plantations have been proposed [18–21]. Significant environmental progress has also been made under the auspices of the Roundtable on Sustainable Palm Oil (RSPO) certification program [22], a result of many of the largest palm oil producers desire to implement environmentally-friendly management.

The Brazilian government is planning a large increase in biofuel production over the next decade, driven by internal and external market demand (ethanol), as well as by government-enforced blending (biodiesel) [23–25]. Much of this expansion is forecast for the eastern Amazonian state of Pará where the palm oil acreage doubled between 2004 and 2010 [25]. Bastos *et al*. [26] suggested that up to 80% of the state would be suitable for oil palm cultivation, with degraded lands (such as abandoned cattle pasture, and mining areas) likely to host much of the growth in production. The Environmental Council of Pará State (COEMA) recently passed a resolution [27] that permits the designation of oil palm as a ‘low-impact’ land-use that may substitute native forest vegetation in the legally-mandated permanent protection areas (áreas de preservação permanente) ‘APPs’ and legal reserves (reservas legais) ‘RLs’ required of smallholder properties of less than 20 ha. With this precedent there is now a powerful rural lobby arguing for this dispensation to be available to all landowners, irrespective of property size [28], as a replacement for regenerating forest vegetation. Given that the forests of the Amazon basin, representing about 41% of the world's remaining tropical rainforest are already subject to deforestation rates fluctuating around half a million hectares per year [29], this expansion into Amazonia requires careful appraisal of the potential impacts of oil palm monocultures on the region’s rich biodiversity. Such impacts have been heavily studied in south-east Asia, where even wildlife-friendly management techniques have failed to conserve species of conservation concern e.g. [30], but there are no such quantitative studies from Amazonia. Decisions about how to balance land requirements for agriculture biofuels and biodiversity conservation will thus have profound effects on the conservation of Amazonian biodiversity, as well as economic development and poverty alleviation [31].

Here we evaluate the value of oil palm plantations for avian biodiversity in relation to other local land-uses (primary and secondary forests and cattle pastures) located in the 145,000 km^2^ Belém Area of Endemism (hereafter Belém AE), a region of eastern Amazonia delimited by the east bank of the Tocantins river and the western biogeographic limit of Amazonian *terra firme* forests in western Maranhão state [32]. Total forest loss in the Belém AE has reached at least 75% of the original extent and further extensive forest loss and concomitant global extinctions are forecast if effective forest conservation policies are not enforced [33], [34]. We investigate the avian biodiversity value of oil palm landscapes in terms of plantation age, tree species richness and distance to source habitats and compare these values with those of land-uses in the surrounding landscape matrix (pastoral systems, primary and secondary forests).

## Materials and Methods

### Study Region

**Fig 1:**
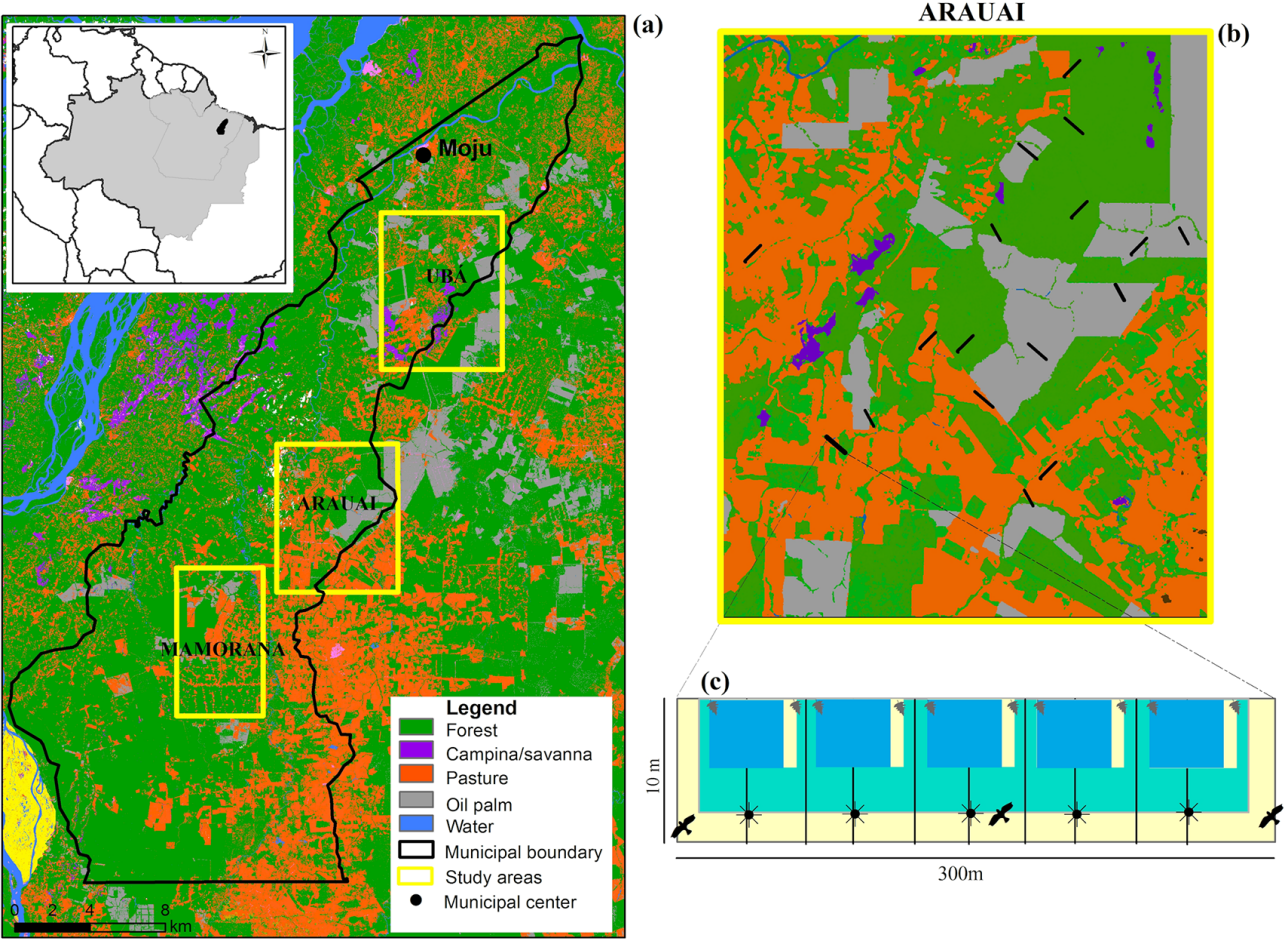
Map of the study region depicting (a) the three areas studied with the municipality of Moju and land-use types; the distribution (b) of transects across one study area (Arauai) and its land-uses; and c) transect design depicting vegetative sampling plots and sub-parcels and the position of point count stations.

This study took place in the municipalities of Moju, Tailândia and Acará located circa 120 km south of Belém in Pará state. Mean annual temperature is 26.6 °C, mean annual precipitation is circa 2,500 mm [35] and local soils are highly weathered acid oxisols [36]. Forest canopy heights are typically in the range of 25–35 m and the understorey is dominated by plants from the families Lecythidaceae, Violaceae, Sapotaceae, Burseraceae, Moraceae and Leguminosae [37]. There are also small patches of natural open vegetation enclaves—*campina* formations on white sand soils which have a very distinct biodiversity [38]. By the year 2010, forest cover in Tailândia and Moju had been reduced by 45% to 4,989 km^2^ much of which is degraded primary or regenerating secondary forest (Fig 1 and [29]). These two municipalities form part of the oil palm ‘pole’ of Moju- Acará- Tailândia, where 124,700 ha of oil palm had been planted by 2009, predicted to increase to 370,500 ha by 2014 [39].

### Data collection

We selected three study regions occupied by different oil palm producers, as follows: Ubá (1,008 km^2^) in the north of the municipality of Moju including the source of the rio Ubá; Arauai (952 km^2^) located in the centre of the municipality including the source of the rio Arauai; and finally Mamorana (680 km^2^) located at the southern extreme of the municipality including the source of the rio Mamorana (for further details see [40]). Between 15–17 300 m transects were allocated to each region, distributed using a stratified-random sampling design (where a standard density of transects (1 per 400 ha) was distributed in proportion to the percentage cover of total forest and production areas) across each region to increase the likelihood that they would capture important internal heterogeneities in forest and/or production systems. To reduce the dependency between transects within each region, transects were separated by a minimum distance of 1.5 km. We also avoided placing transects within 200 m of any other land-use to control for potential edge effects.

In total we sampled 50 transects, 17 in primary forest, 4 in secondary forests—forests that developed after complete deforestation [41] of different ages (5, 7, 15 & 20 years old), 15 in variable-age oil palm plantations (1, 3, 4, 12, 14, 23, 24 & 25 years old),12 in cattle pasture and one in *campina* vegetation (although the latter was not used in later analyses except Fig. C in S1 File). Designation of secondary forest and plantation ages was done through visual inspection of a 20 year time-series of Landsat images for each transect, calibrated by interviews with local farmers. All primary forest sites had been subject to historic selective logging events but none have been burned in the last five years. All fieldwork took place on private lands and landowners in each region were visited prior to any fieldwork to introduce the project and secure permissions for surveys. Land-use classification was undertaken using ArcGis 9.3 and Envi 4.5 using both unsupervised (ISODATA) and supervised (MaxVer) classification protocols (ISODATA) [40]. The ISODATA (Interactive Self-organizing Data Analysis) method was used to determine land-use classes prior to fieldwork and employed minimum Euclidean distance measurements (in multidimensional space) to identify spectral clusters from the image and assign a class to each pixel. On returning from the field with our ground-truthed measurements we performed a supervised MaxVer (Maximum Likelihood Classification) analysis which assigns the probability of each pixel to pre-defined classes (based on our ground-truthing), assigning pixels to the class with the highest probability. To assess classification accuracy we used Cohen’s Kappa statistic [42], which provides an indication of the classification agreement between the classified and the ground-truthed maps that is not attributable to chance. The remote sensing analysis was performed using georeferenced data with Landsat TM-5 (Thematic Mapper) images with 30 m spatial resolution from the year 2010.

To sample the bird community we located three point count (PC) stations in each transect at 0, 150 and 300 m. A total of 288 PCs were conducted between the three regions. We (ACL & NGM) carried out two repetitions of three 15 minute, 75 m fixed radius PCs per transect, recording all species seen or heard. Repetitions ensured that temporal variation in avian vocal activity was minimized, and PCs were recorded using solid state recorders. The avifaunal surveys were complemented with sampling of woody plants and vegetative structure to see how habitat structural characteristics and botanic diversity might influence avian species richness. These surveys targeted trees and lianas and palms above 2 cm DBH (Diameter at Breast Height) and were conducted in 10 x 250 m plots, subdivided into 10 x (10 x 15 m) parcels following the protocols of Gardner et al. [43]. All individuals were identified to species or morphospecies by expert parabotanists (Nelson Rosa and Carlos Alberto Santos). Plant species which defied field identification were collected and deposited in the herbarium of the Museu Paraense Emílio Goeldi for later identification. To generate biomass estimates (as Mg.ha-^1^) we used allometric equations for all plant species with DBH ≥ 2 cm.

We used the equations of Chave et al. [44] for humid forest tree species:

$$\begin{array}{l} \left(\text{AGB}\right)\text{est}=\text{p}\;\times \text{exp}(-1.499\;+2.1481\;\times ln\left(DBH\right)\;+0.207\;\times {\left(ln\left(DBH\right)\right)}^{2}\;-0.0281\\ \;\times \;{\left(ln\left(\text{DBH}\right)\right)}^{3} \end{array}$$

where: AGB = above ground biomass for all species with DBH ≥ 2 cm; p = wood density (g/cm-3); and DBH = diameter at breast height (cm). We used a genera-specific equation for palms in the genus *Cecropia*, that of Nelson et al. [45]:

DW=exp(−2.5118+2.4257×ln(DBH))

where DW = estimated biomass of *Cercropia* sp. trees. Finally to generate estimates of the biomass of oil palm *Elaeis guineensis* we used the age-specific formula of Corley & Tinker [46]:

$$\text{Bt}=\pi \;\times \left(\text{r}\;\times \;{\text{Z}}^{2}\right)\times 100\times \text{h}\;\times \rho$$

where Bt = estimated biomass (kg) of *Elaeis guineensis*; r = radius (cm) of the palm’s trunk; Z = diameter of the palm base (a constant—0.777), ρ = height (m) of the trunk and ρ = trunk density (Kg/m^3^) which is calculated using the formula:

$$\rho =\frac{\text{Id}\times 0.0076+0.083}{1000}$$

Where Id is the age of the oil palm in years.

### Data analyses

We analysed the responses of total species richness as well as richness and turnover for the subset of ‘primary forest-associated birds’ (hereafter termed ‘forest birds’). These forest birds represent the core avifauna of undisturbed *terra firme* forests but not necessarily birds restricted to those habitats, as some core primary forest species also occur in human-modified forest and non-forest habitats (e.g. Blue-gray Tanager *Tangara episcopus* and Bananaquit *Coereba flaveola*). These categorizations were based on previously published classification of birds from the region e.g. [47] and [48]. Our taxonomy follows the checklist of Brazilian birds compiled by the Comitê Brasileiro de Registros Ornitológicos [49].

To compare sampling intensity and avian responses between different land-uses we used sample-based rarefaction curves, with 95% confidence intervals calculated using the ‘specaccum’ tool, included in the vegan package of the R software [50]. Comparisons between species richness in each land-use type were made using a one-way ANOVA test with intervals followed by a Tukey *post-hoc* test to check for significant pairwise differences. To explore univariate relationships between forest cover and forest bird species richness we performed linear regressions using percentage of total forest cover (primary and old (>15 years) secondary forests combined in a 1 km buffer), the percentage of primary forest cover in a 1 km buffer around the centroid of each transect (to standardise sampling unit size) and distance to the nearest primary forest as predictor variables for avian species richness.

To assess the relative importance of different environmental variables on bird species richness we first assessed for variable colinearity using Pearson correlation between the variables, edge distance, tree species richness, plants biomass and percentage of primary forest cover excluding variables with an unacceptably-high degree of colinearity (r ≥ 0.7). We then used generalized linear models (GLMs) with a Gaussian distribution and a log link function and model averaging using AICc weights [51] to generate subsets of top models and uncover the relative importance of different variables. All these analysis were done using R version 2.15.1 [50] with the ‘glmulti’ and ‘MuMin’ packages.

To assess the variation in species composition between land-use systems and different primary forest disturbance classes we produced non-metric multi-dimensional scaling ordinations (nMDS [52]) using the Sorensen similarity matrix for species presence-absence data. To assess the statistical significance of differences in assemblage composition between different land-use types and forest degradation classes we conducted a one-way PERMANOVA which uses pseudo-F values to compare among-group to within-group similarity and assesses significance by permutation. All multivariate assemblage analyses were carried out in Primer v.6 (PRIMER-E Ltd, Plymouth, UK, [53]). To facilitate comparison to a larger sample of different land-uses we compared our data from Moju-Tailândia-Acará with data from an extensive inventory of the neighbouring municipality of Paragominas [41]. This inventory used the same transect selection and avian sampling protocols and has the same source avifauna [54], [55] as the present study and was conducted by the same field ornithologists (ACL & NGM). In total we used avian data from 187 transects—97 in primary forest, 25 in secondary forest, 53 in cattle pasture and 12 from mechanised agriculture (soy bean fields in this case). We investigated relative contribution of individual species to the overall dissimilarity using the similarity percentages routine-SIMPER [50]. Owing to historic legacy effects of land purchasing (with smallholder properties typically closer to forest borders), oil palm plantations were on average situated farther from primary forest borders (mean = 972 m, SD = 267 m, range 545–1424 m) than cattle pastures (mean = 510 m, SD = 150 m, range 216–719 m). To control for this potential bias of leakage of forest species we compared oil palm forest bird richness with forest bird richness from cattle pastures (n = 23 mean = 1009 m, SD = 382 m, range 554–2069 m) in Paragominas situated over 550 m from the nearest primary forest border and assessed the significance of differences with a paired t-test using R.

Finally, we used a global phylogeny of birds [56] to visually compare the phylogenetic structure of the most speciose avian clades (families with >8 species) between the dominant regional land-uses (primary forest, pasture and oil palm). The resulting circular phylograms were visualized and edited using the FigTree v 1.4.1 software (http://tree.bio.ed.ac.uk/software/figtree/) to document non-random extinction processes.

## Results

### Regional land-use classification

Across the 2,588 km^2^ study region our remote-sensing analysis identified seven discrete land-use types: varyingly-degraded primary forest: 40.2% (1041 km^2^), secondary forest: 9.0% (234.9 km^2^), *campina* formations: 2.8% (71.8 km^2^), oil palm plantations: 8.3% (214.9 km^2^), cattle pasture: 39.2% (1016 km^2^), open water: 0.3% (9.0 km^2^) and cloud/shadow: 0.01% (0.15 km^2^). These results were associated with a Kappa coefficient of 0.85 (with a global accuracy of 89% of the pixels correctly classified). Following Monserud and Leemans [57] Kappa statistics values, from 0.7 to 0.85 represent very good agreement between images.

### Species richness between land-use types

**Fig 2:**
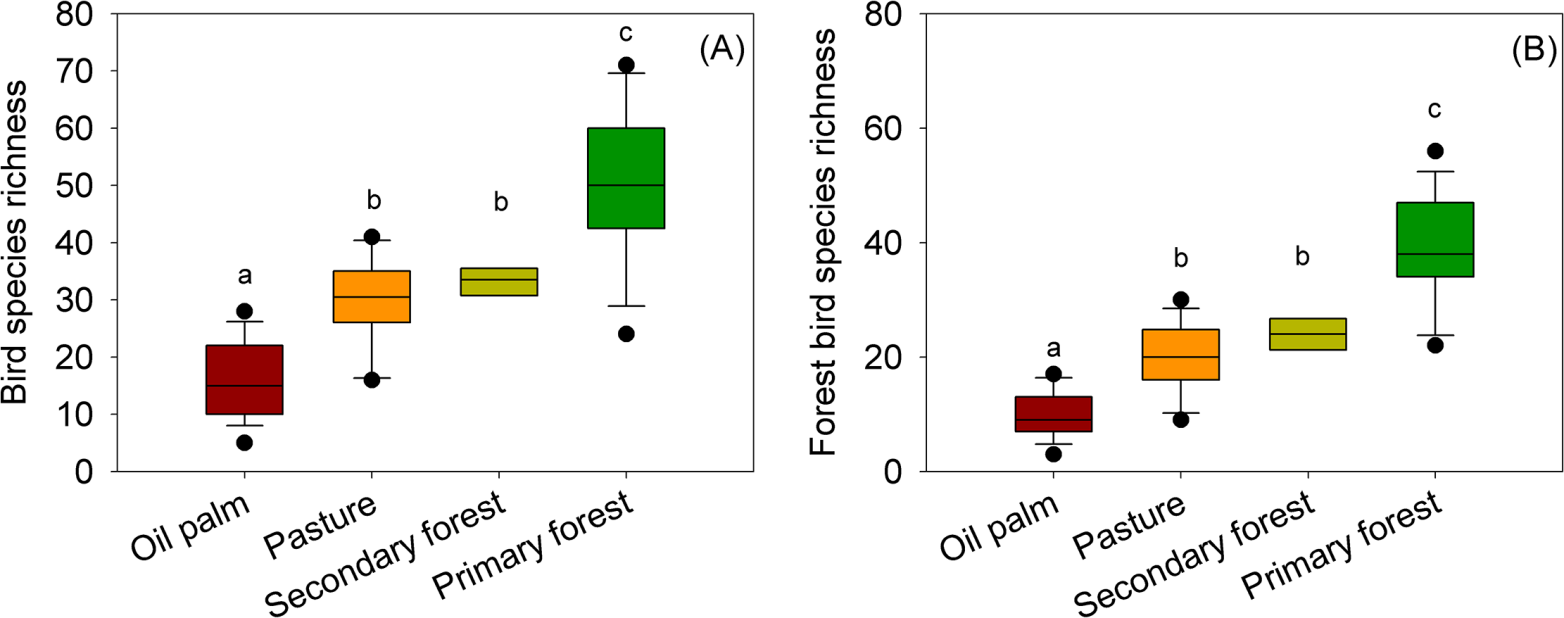
Box plots comparing avian species richness between land-use types, using the entire avian assemblage (A) and just forest-associated birds (B). Non-significant pairwise differences between land-use types are indicated by the presence of the same letter (according to a Tukey test 95%).

We recorded 3,090 detections of 249 bird species, of which 1982 were forest-associated species (for a full list see Table A in S1 File). The species accumulation curves indicated that surveys in most land-use types were near asymptotic (Fig A in S1 File). We recorded mean species richness per transect of 50.1 in primary forest (SD = 13.2, n = 16, total richness = 211, range = 24–69,), 33.25 in secondary forest (SD = 2.3, n = 4, total richness = 68, range = 30–36), 30.0 in cattle pasture (SD = 7.9, n = 12, total richness = 100, range = 17–41), and 16.3 in oil palm plantation (SD = 6.9, n = 15, total richness = 69, range = 6–28). These differences in species richness were significant between all land-use types considering the whole avifaunal community (Fig 2A, F = 32; df = 43; N = 4; P< 0.01) and for forest-associated birds (Fig 2B, F = 46; df = 43; N = 4; P< 0.01), with the exception of those between pasture and secondary forests for which the mean differences richness per transect was statistically non-significant (Fig 2). The difference in forest bird species richness was not significant between oil palm and cattle pastures (mean = 4.34, SD = 2.19, n = 23) situated over 500 m from the nearest primary forest in Paragominas (unpaired t-test, t = 1.7; df = 36; P = 0.09, Fig. C, in S1 File). We found that avian species was statistically different between old (>11 years, n = 6, mean species richness: 10.2) and young (<10 years, n = 8, mean species richness: 21.5) oil palm transects for both the entire avian community (F = 29.4; df = 13; P<0.01) and forest-associated birds only (F = 18.7; df = 13; P<0.01). Furthermore, avian communities in oil palm plantations of varying ages underwent avian community succession resulting in different community structure and richness between plantations of different ages (Fig C, in S1 File). Recently planted stands (≈ 1–2 years) had a very low vegetative biomass and were mostly occupied by birds typical of cattle pastures (or natural savannah enclaves—see [38]) such as Pale-breasted Spinetails *Synallaxis albescens* and Red-breasted Blackbirds *Sturnella militaris*. Plantations of ≈ 5 years had a more significant biomass comparable with young secondary forests, but had a similar community structure, albeit including some species more typical of edge/secondary forest such as Reddish Hermit *Phaethornis ruber*, Great Antshrike *Taraba major* and White-fringed Antwren *Formicivora grisea*.

### Environmental determinants of species richness

**Fig 3:**
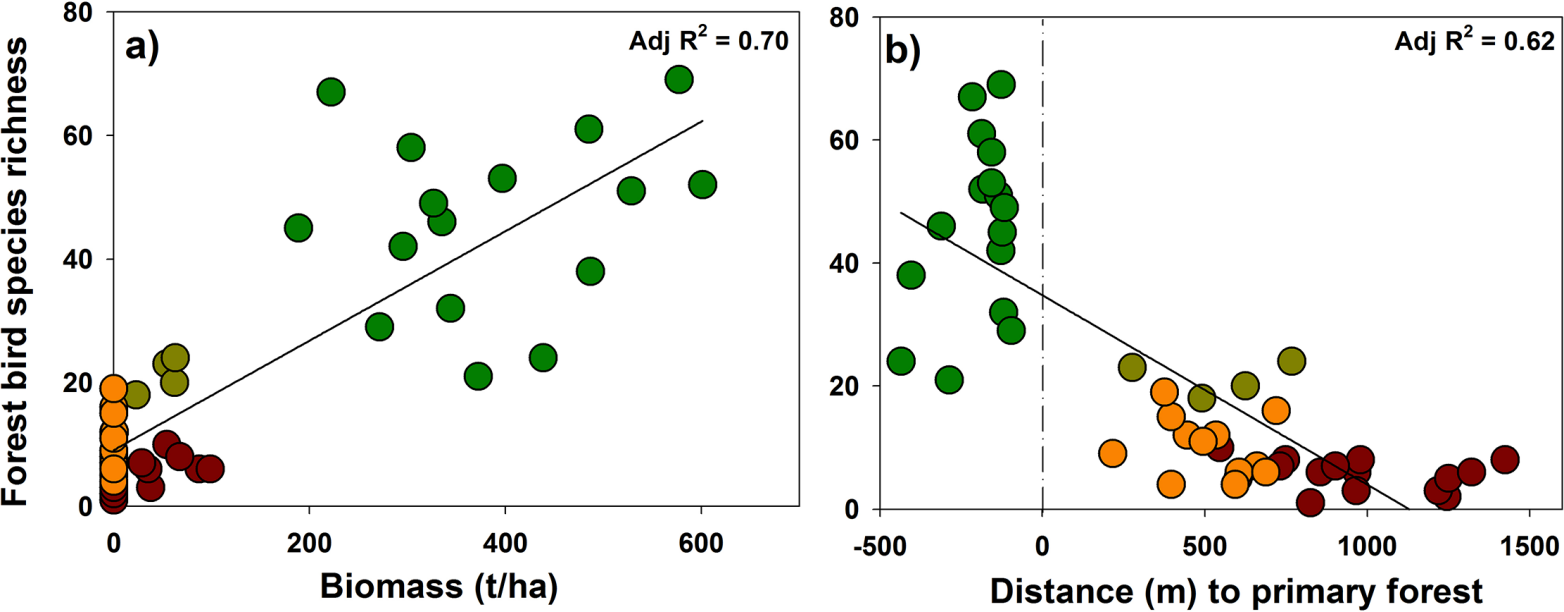
Linear regressions between richness of forest bird species and a) tree biomass and b) distance to the nearest primary forest border.

**Fig 4:**
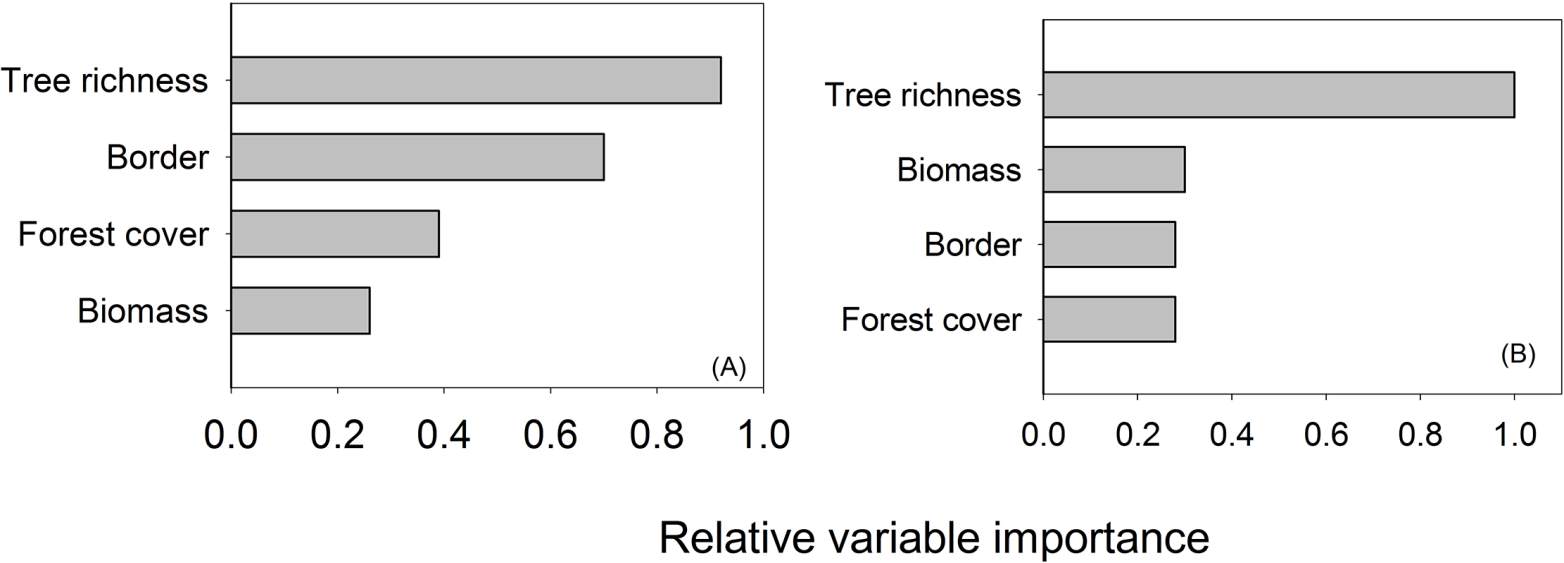
Relative importance of environmental variables in explaining total avian species richness (A) and forest-associated bird richness (B). Signs indicate the direction of the effect of each variable. The explanatory variables include distance to the forest border (border), tree species richness, forest cover (% of primary forest cover) and biomass of trees (biomass).

We found a significant positive and broadly linear relationship between the richness of all bird species and tree biomass (adj. R^2^ = 0.49, p < 0.001) which strengthened when only forest associated birds were included in the model (Fig 3, adj. R^2^ = 0.70, P < 0.001). We uncovered a significant negative relationship between distance to forest edge and species richness (adj. R^2^ = 0.56, P < 0.001) which was marginally more significant when only forest birds were retained (Fig 3, adj. R^2^ = 0.56, P < 0.001). The GLMs revealed that tree richness was the most important predictor variable influencing richness for the whole community (Fig 4 and Table B in S1 File) followed by distance to the forest border, forest cover and vegetative biomass for the whole community, whilst for forest associated birds the most important predictor variables were tree species richness, biomass, distance to the forest border and forest cover. The top model results showed that, considering the whole avian community, four models had low ΔAICc (<2), with the best model including distance to border and tree richness. Considering only forest-associated birds two models had low ΔAICc with the best retaining only tree species richness (Table B in S1 File).

### Avian community structure across land-use types

**Fig 5:**
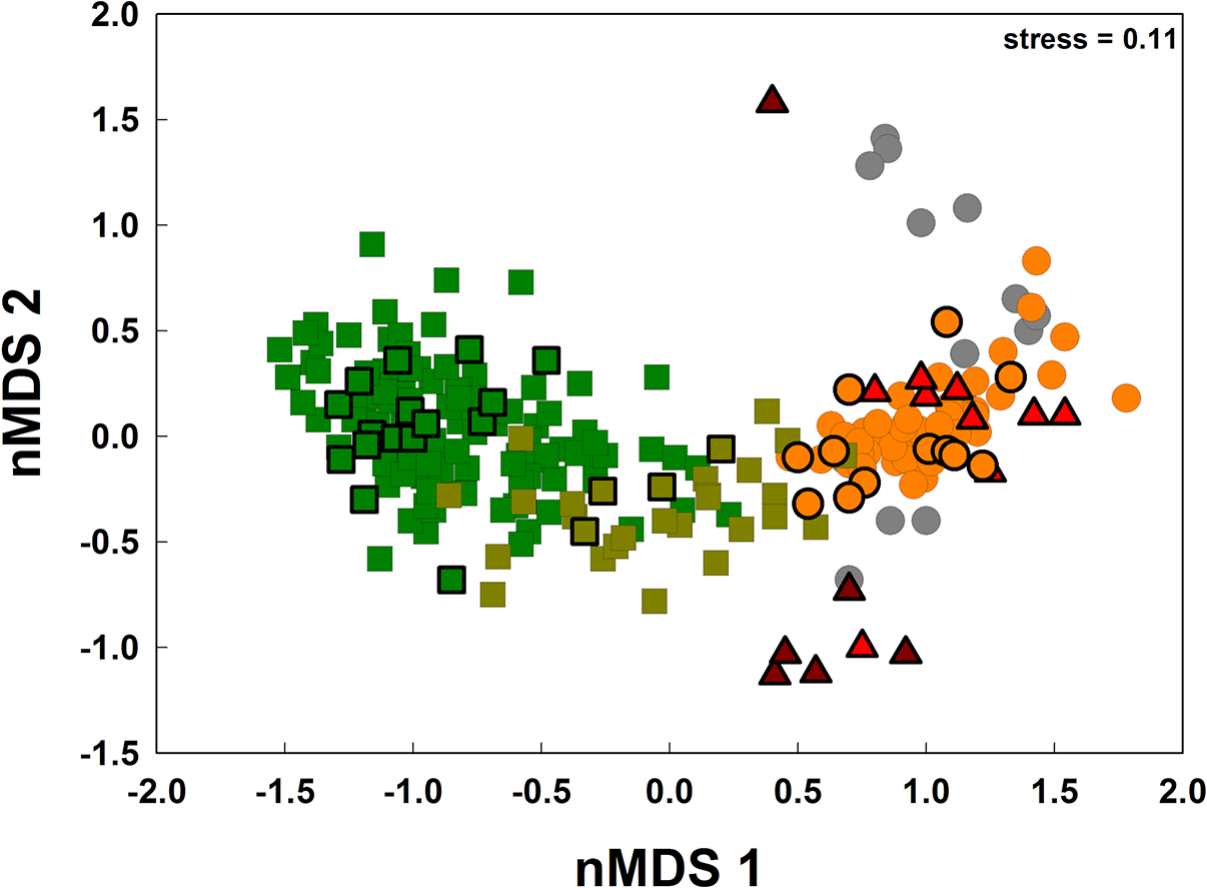
NMDS plots of community structure of the avian community in Moju (polygons with heavy black borders) and Paragominas (narrow borders), primary forest transects are represented by dark green squares, secondary forests by light green squares, orange circles are cattle pastures, grey circles are mechanised agriculture and triangles are oil palm plantations (dark red = 15–20 years old, lighter red 0–6 years old).

**Fig 6:**
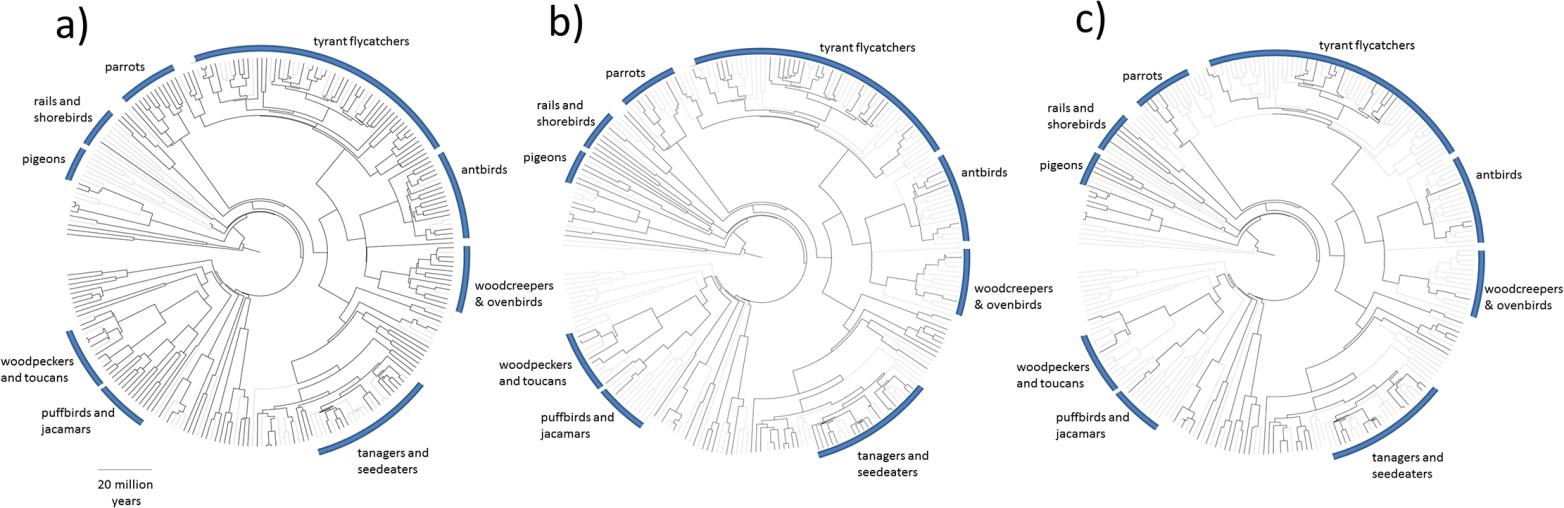
Circular phylograms illustrating avian community composition in a) primary forests b) cattle pasture and c) oil palm plantations, families with more than eight species are labelled. Bold lines indicate species presences in the given land-use type whereas pale lines denote species absences from that land-use type which were found in one or more other land-uses.

Avian species composition changed consistently and significantly along a gradient of human impacts between primary forests, secondary forests, cattle pastures and oil palm plantations Fig 5, Table C in S1 File, PERMANOVA, Pseudo-F = 8.1725, P < 0.001). All species assemblages were significantly different from each other (P < 0.001) with the exception of pastures and plantations for which P = 0.573. Community structure in primary forests, secondary forests and cattle pasture was broadly similar to that found in the neighbouring municipality of Paragominas (Fig 5) indicating low species turnover between the municipalities and emphasising the generalizability of the results across the Belém AE. Each land-use type played host to characteristic assemblages of species whose relative dominance contributed to community dissimilarity (Table 1), with communities in the oil palm plantations sharing more species with cattle pastures than they did with forest habitats. The phylograms (Fig 6) indicate that land-use mediated local extinctions were non-random, with greatest losses of species occurring from the diverse radiation of suboscine passerines (antbirds, tyrant flycatchers, ovenbirds and woodcreepers) for which few species persisted in cattle pastures or oil palm plantations. There was some turnover of species, with waterbirds such as crakes and rails and diverse granivorous oscine passerines typical of open areas colonising the pastures and oil palm plantations (Fig 6. Table A in S1 File).

**Table 1:** The top-ranked 15 bird species in each land-use type which contributed to the dissimilarity of species composition between oil palm plantations, cattle pastures, secondary forests and primary forests.

**Oil Palm**				**Pasture**			
Latin binomial	Av.Abund	Contrib%	Cumu.%	Latin binomial	Av.Abund	Contrib%	Cumu.%
*Formicivora grisea* ^*1*^	1	20.7	20.7	*Myiophobus fasciatus* ^*2*^	0.92	7.54	7.54
*Tyrannus melancholicus*	0.8	11.89	32.59	*Tangara episcopus*	0.92	7.54	15.09
*Ramphocelus carbo* ^*4*^	0.67	8.33	40.92	*Tyrannus melancholicus*	0.92	7.54	22.63
*Tangara palmarum*	0.53	5.48	46.41	*Troglodytes musculus* ^*2*^	0.85	6.4	29.03
*Myiophobus fasciatus* ^*2*^	0.6	5.19	51.6	*Elaenia flavogaster*	0.85	5.97	35
*Troglodytes musculus* ^*2*^	0.53	4.76	56.36	*Phaeomyias murina*	0.85	5.97	40.97
*Pitangus sulphuratus*	0.47	3.79	60.15	*Ramphocelus carbo* ^*4*^	0.85	5.41	46.38
*Turdus leucomelas*	0.4	3.48	63.62	*Synallaxis albescens* ^*2*^	0.69	4.14	50.52
*Phaethornis ruber* ^*3*^	0.4	3	66.63	*Tangara palmarum*	0.69	4.06	54.58
*Sturnella militaris* ^*2*^	0.47	2.93	69.56	*Volatinia jacarina* ^*2*^	0.69	3.93	58.51
*Volatinia jacarina* ^*2*^	0.47	2.93	72.49	*Sturnella militaris* ^*2*^	0.62	3.64	62.15
*Progne chalybea* ^*3*^	0.4	2.68	75.17	*Formicivora grisea*	0.62	2.82	64.97
*Synallaxis albescens* ^*2*^	0.4	2.11	77.28	*Progne chalybea* ^*3*^	0.54	2.27	67.24
*Crotophaga ani*	0.4	2.02	79.3	*Tachyphonus rufus*	0.54	2.26	69.5
*Amazona amazonica*	0.33	1.78	81.09	*Myiarchus tyrannulus*	0.54	2.17	71.67

**Secondary Forest**				**Primary Forest**			
Latin binomial	Av.Abund	Contrib%	Cumu.%	Latin binomial	Av.Abund	Contrib%	Cumu.%
*Coereba flaveola* ^*2*^	1	6.11	6.11	*Cercomacra cinerascens*	1	4.54	4.54
*Formicivora grisea*	1	6.11	12.22	*Lophotriccus galeatus*	1	4.54	9.09
*Phaethornis ruber* ^*3*^	1	6.11	18.34	*Phaethornis ruber* ^*3*^	0.93	4.04	13.13
*Pheugopedius genibarbis*	1	6.11	24.45	*Myrmotherula axillaris* ^*2*^	0.93	3.97	17.1
*Progne chalybea* ^*2*^	1	6.11	30.56	*Pyriglena leuconota* ^*2*^	0.93	3.94	21.03
*Pyriglena leuconota* ^*2*^	1	6.11	36.67	*Glyphorynchus spirurus*	0.87	3.32	24.36
*Ramphocelus carbo* ^*4*^	1	6.11	42.78	*Myiopagis gaimardii* ^*2*^	0.8	2.97	27.33
*Thamnophilus amazonicus*	1	6.11	48.89	*Pheugopedius genibarbis*	0.8	2.78	30.11
*Amazona amazonica*	0.75	3.14	52.04	*Ramphocelus carbo* ^*4*^	0.8	2.78	32.88
*Manacus manacus*	0.75	3.14	55.18	*Thamnomanes caesius*	0.8	2.62	35.5
*Patagioenas speciosa*	0.75	3.14	58.32	*Isleria hauxwelli*	0.73	2.25	37.75
*Saltator maximus*	0.75	3.14	61.46	*Coereba flaveola* ^*2*^	0.73	2.22	39.97
*Lophotriccus galeatus*	0.75	3.08	64.53	*Zimmerius gracilipes*	0.73	2.15	42.12
*Myiopagis gaimardii* ^*2*^	0.75	3.08	67.61	*Thamnophilus amazonicus*	0.67	2.11	44.23
*Myrmotherula axillaris* ^*2*^	0.75	3.08	70.69	*Ramphastos vitellinus*	0.67	1.89	46.12

Numbered superscripts refer to the number of other habitats in which species were also top-ranked contributors to species similarity.

### Occurrence of threatened and endemic species

Most of the bird species (96%) we detected are classified as Least Concern by BirdLife International [58] with the exception of nine species in the threat categories Endangered (EN), Vulnerable (VU) and Near Threatened (NT). These were as follows: White-crested Guan *Penelope pileata* (VU); Ruddy Pigeon *Patagioenas subvinacea* (VU); Golden Parakeet *Guaruba guarouba* (VU); White-bellied Parrot *Pionites leucogaster* (EN); Pearly Parakeet *Pyrrhura lepida* (VU); Vulturine Parrot *Pyrilia vulturina* (VU); (Eastern) Red-necked Aracari *Pteroglossus bitorquatus* (EN); Red-billed Toucan *Ramphastos tucanus (*VU); Channel-billed Toucan *Ramphastos vitellinus* (EN) (as Ariel Toucan *Ramphastos ariel*) and Long-tailed Woodcreeper *Deconychura longicauda* (NT). All these species were confined to primary forests; with the exception of a single Red-necked Aracari photographed in an arborescent pasture [59] and one record of Channel-billed Toucan from a secondary forest transect (Table A in S1 File). In addition we found a further four species which are narrowly endemic to the Belém AE (and a further four highly-differentiated endemic subspecies likely to be subject to future taxonomic upgrades to species status [60–62]) and six other species that are restricted to south-east Amazonia, east of the river Madeira and south of the river Amazon. We also recorded an undescribed species of *Myiornis* pygmy-tyrant from one primary forest transect, which is now known from several forest sites in north-east Brazil, see [38].

## Discussion

We found that oil palm plantations in eastern Amazonia supported species-poor avian communities of comparable richness and composition to other non-forest land-uses such as cattle pasture. These communities in oil palm were principally composed of non-forest species of low conservation concern. This general conclusion is supported by results from similar studies of tropical oil palm plantations in formerly forested landscapes in Borneo [30], Peninsula Malaysia [63], Thailand [64], and Colombia [65]. Furthermore, we expect that the loss and turnover of the avian community in our landscape will be mirrored in diverse other taxonomic groups (e.g. mammals, reptiles, invertebrates). Responses of forest bird communities to oil palm plantations have been shown to be an excellent indicator of responses in other taxonomic groups in other countries [30], [66], [67] and forest birds have been shown to be excellent indicators of the responses of diverse taxonomic groups to other forms of land-use change in Amazonia [68]. As such, we consider that oil palm plantations cannot be considered to be ‘low impact’ land-uses and reinforces the current position of the COEMA [28] in not permitting oil palm to be used a substitute for native vegetation in APPs or RLs for large property owners.

### Avian biodiversity in Amazonian oil palm plantations

We did not find any species of conservation concern within the plantations nor any species typically regarded as being indicators of *terra firme* forest habitats. Species occupying oil palm plantations were typically a subset of those that occupy other Amazonian non-forest land-uses (e.g. [55]). These communities are dominated by a small number of generalist species which disproportionately contribute to community dissimilarity from other land-uses. Likewise we did not find evidence for positive effects of proximity to large forest tracts for within-plantation biodiversity, although we avoided habitat edges by at least 200 m to control for inflation of species richness through spillover [68]. Although oil palm may be of low value for most forest birds, birds may be of value to oil palm producers, through insectivorous passerine top-down control of phytophagous insect herbivory and through predation on rodents by raptors [69]. We frequently encountered species like White-tailed Hawks *Geranoaetus albicaudatus* hunting in the plantations and local population densities of this and other raptors might be increased through provision of nest boxes and hunting perches.

Although we generally found avian communities in oil palm plantations to be typical of anthropogenic land-uses, a notable exception to this rule came from two transects which were planted on what was originally open habitat *campina* formations (rather than humid moist forest). These transects hosted some species regionally restricted to these non-forest enclaves e.g. Ocellated Crake *Micropygias chomburgkii*, Rusty-backed Antwren *Formicivora rufa* and Lesser Elaenia *Elaenia chiriquensis*, which we also encountered at several other non-degraded *campina* enclaves [38]. Owing to the poor sandy soil at these localities the palms were stunted and some of the original *campina* physiognomy had been retained between the rows of palms. The inclusion of these transects resulted in an inflation of the total richness in oil palm plantations and we note that *campina* formations are considered to be “Zonas Ambientalmente Sensíveis” (Environmentally sensitive areas), which may only be used with the adoption of technologies and with an intensity compatible with local environmental conditions [70].

Despite their higher basal area and presence of epiphytes we found older plantations (<15 years) to be even more depauperate than younger plantations, most of the species typical of pastures vanish, with a closed canopy leaving very few species typical of more arborescent (but still edge) habitats, such as Yellow-breasted Flycatcher *Tolmomyias flaviventris* and Gray-chested Greenlet *Hylophilus semicinereus* and abundant White-fringed Antwrens and Pale-breasted Thrushes *Turdus leucomelas*.

### Avian biodiversity in the remaining forest matrix

Although we consider oil palm plantations to be very poor habitat for Amazonian forest bird species, we did however find significant avian biodiversity within the remaining skeletal forest matrix of the region. These included species of national and global conservation concern such as Golden Parakeet and Vulturine Parrot. Fieldwork by other ornithologists in the same fragments has even resulted in confirmation of breeding of top predators such as Harpy Eagle *Harpia harpyja* [71]. However, even the avifauna within these forest fragments represents a shifted avian biodiversity baseline as many forest-dependent species previously recorded by our surveys in high basal area transects in neighbouring Paragominas [53] such as Snethlage's Antpitta *Hylopezus paraensis*, Opal-crowned Manakin *Lepidothix iris iris* and Tawny-crowned Greenlet *Hylophilus ochraceiceps rubrifrons* were unrecorded in Moju-Tailândia both by our surveys and those of Silveira [72]. We infer their local extinction given their historical disappearance from degraded primary forests elsewhere in the region [73] and attribute this loss to habitat modification from selective logging and/or fire which afflicted these forest patches before they became established as legal forest reserves (RLs: Reservas Legais) by the oil palm growers. If connectivity to existing undisturbed forest nuclei can be restored, either by making sure that obligations to maintain APPs are fulfilled or setting aside conservation corridors, then it is possible that some of these disturbance-sensitive taxa may recolonize the forest fragments [74]. However, application of the new Brazilian Forest Code will result in the loss of ≈ 60% of forest vegetation from the APPs in the region [40].

We recorded very few game birds such as tinamous and cracids in the forest fragments and from this infer that these patches are subject to heavy hunting pressure. This assumption is backed up by regular encounters with human hunters and detection of hunter artefacts (trails, shot gun cartridges, elevated hunter ‘perches’). Large-bodied fauna is nominally protected in these areas by vehicle checkpoints and patrols operated by the oil palm companies and these initiatives by the companies are to be applauded and encouraged. However, although these measures may act as a significant deterrent for some hunters, policing such large areas is obviously extremely difficult. This effect of protection was most noticeable for *Sporophila* seedeaters which were recorded commonly within area managed by oil palm companies but very rarely outside due to widespread trapping of these species for the wild bird trade.

### Implications of oil palm expansion for regional biodiversity

In comparison with an exhaustive survey of regional land-uses we do not consider oil palm to be of any greater value to birds than other land-uses in Amazonia such as cattle pasture, soybean and eucalyptus plantations (e.g. [55], [67], [75]). These conclusions are echoed throughout the world in studies of forest monocultures (e.g. [76], [77]). It is possible that oil palm may function as a more permeable matrix for some bird species than other non-forest land-uses such as soy bean plantations and this hypothesis merits testing with dispersal challenge experiments or radio-tagging experiments, although we note that recent research indicates that even secondary forest is an effective barrier to dispersal for many primary forest species [78].

Despite the poor habitat value for Amazonian biodiversity, we recognize that oil palm may be an important alternative for regional development given its positive role in the potential recovery of abandoned areas, income generation and in producing renewable energy [5], [6], [46]. However, sustainable production of palm oil must include solid promises that any expansion growth does not come at the expense of existing forest habitats through direct or indirect deforestation [79] in accord with Ecological-Economic Zoning initiatives [26], [70].There should be ample room for expansion on degraded pastures without putting pressure on existing forests, including secondary forests in early successional stages [80]. For example, data from the TerraClass [81] initiative reveals that there are 9.6 million hectares of abandoned pastures in the Legal Amazon of which 3.3 million are in the state of Pará. In contrast, there is only one strictly protected area in the Belém AE, the beleaguered Reserva Biológica do Gurupi, which protects just 1.4% of the land area in this biogeographic province [82]. As a consequence even small and medium-sized forest remnants in this region have high global conservation value.

Moreover, given the relatively strict Brazilian environmental regulations associated with the Forest Code there is potential to lever oil palm environmental standards globally through inter-continental competition for the ‘green’ oil palm market under the auspices of the RSPO. International pressure to comply with Brazil’s strict environmental minimal standards might benefit Amazonian biodiversity if oil palm companies prove to be better stewards of the remaining forests than other regional actors and participate in schemes to secure the long-term future of existing forests and improve landscape-level connectivity.

## Supporting Information

S1 File This contains Tables A, B, and C and Figures A, B, and C. Table A. Systematic list (following CBRO 2014) of bird species recorded in the land-uses: PF = primary forest, SF = secondary forest, CP = cattle pasture, OP = oil palm. The status column highlights both their global Red List status following Birdlife International (2014), where VU = Vulnerable and EN = Endangered and their endemicity (following HBW 2015), where END1 = full species endemic to the Belém AE (and adjacent forests of a similar physiognomy in north-east Brazil), END1* = subspecies endemic to the Belém AE and END2 = species endemic to south-east Amazonia, west of the river Madeira and south of the river Amazon. **Table B.** Top ranked model results from GLMs for the whole avian community and forest birds alone. The explanatory variables include distance to the forest border (Border), tree species richness (Tree richness), forest cover (% of primary forest cover) and biomass of trees (Biomass). For each model R^2^ is the proportion of variation explained, ΔAICc is the difference between AICc between this and the preceding model and weight is the Akaike weight for the given model. **Table C.** PERMANOVA Pseudo-F statistic values of the global test and P-value and t values of pair-wise comparison, P-values and mean similarity of bird community composition in different land-use types. **Fig. A.** Species rarefaction curves per point count considering the entire avian assemblage in land-uses (A) primary forest, (B) secondary forest, (C) cattle pasture & (D) oil palm. **Fig. B.** Relationship between distance to the nearest primary forest border and richness of forest bird species for nom primary forest transects around Moju (heavy dark border) and Paragominas (narrow dark border). Green circles denote secondary forest transects, orange circles = cattle pasture, grey circles = mechanised agriculture and red circles = oil palm. Oil palm transects have a comparable species richness to other non-forest land-uses. **Fig. C.** nMDS plot of community structure of the entire avian assemblage in Moju, primary forest transects are represented by dark green squares, secondary forests by light green squares, cattle pastures are yellow circles, the blue star is a natural *campina* formation, dark red triangles are older oil palm plantations (12–25 years), lighter red triangles are intermediate aged oil palm plantations (3–4 years) and orange triangles are recently-planted oil palm plantations (1–2 years). Polygon size is proportional to species richness.
(DOCX)
