# Supplementary material for: Poor Prospects for Avian Biodiversity in Amazonian Oil Palm
Source: PLoS One. 2015 May 8;10(5):e0122432. doi: 10.1371/journal.pone.0122432 (PMC4425670; doi:10.1371/journal.pone.0122432)

**Supplementary Material 1**

**Table A.** Systematic list (following CBRO 2014) of bird species recorded in the land-uses: PF = primary forest, SF = secondary forest, CP = cattle pasture, OP = oil palm. The status column highlights both their global Red List status following Birdlife International (2014), where VU = Vulnerable and EN = Endangered and their endemicity (following HBW 2015), where END1 = full species endemic to the Belém AE (and adjacent forests of a similar physiognomy in north-east Brazil), END1* = subspecies endemic to the Belém AE and END2 = species endemic to south-east Amazonia, west of the river Madeira and south of the river Amazon.

| **Taxonomic classification & latin binomial** | **English Name** | **Status** | **PF** | **SF** | **CP** | **OP** |
| --- | --- | --- | --- | --- | --- | --- |
| Tinamiformes Huxley, 1872 |  |  |  |  |  |  |
| Tinamidae Gray, 1840 |  |  |  |  |  |  |
| *Crypturellus soui* (Hermann, 1783) | Little Tinamou |  | 1 | 1 |  |  |
| *Crypturellus variegatus* (Gmelin, 1789) | Variegated Tinamou |  | 1 |  |  |  |
| Anseriformes Linnaeus, 1758 |  |  |  |  |  |  |
| Anatinae Leach, 1820 |  |  |  |  |  |  |
| *Cairina moschata* (Linnaeus, 1758) | Muscovy Duck |  |  |  | 1 |  |
| Galliformes Linnaeus, 1758 |  |  |  |  |  |  |
| Cracidae Rafinesque, 1815 |  |  |  |  |  |  |
| *Penelope pileata* Wagler, 1830 | White-crested Guan | VU | 1 |  |  |  |
| *Ortalis superciliaris* (Gray, 1867) | Buff-browed Chachalaca | **END1** |  |  | 1 |  |
| Pelecaniformes Sharpe, 1891 |  |  |  |  |  |  |
| Ardeidae Leach, 1820 |  |  |  |  |  |  |
| *Butorides striata* (Linnaeus, 1758) | Striated Heron |  |  |  | 1 |  |
| Cathartiformes Seebohm, 1890 |  |  |  |  |  |  |
| *Cathartes aura* (Linnaeus, 1758) | Turkey Vulture |  | 1 |  | 1 | 1 |
| *Cathartes melambrotus* Wetmore, 1964 | Greater Yellow-headed Vulture |  | 1 |  |  |  |
| *Coragyps atratus* (Bechstein, 1793) | Black Vulture |  |  |  | 1 | 1 |
| Accipitriformes Bonaparte, 1831 |  |  | 1 |  |  |  |
| Accipitridae Vigors, 1824 |  |  | 1 |  |  |  |
| *Leptodon cayanensis* (Latham, 1790) | Gray-headed Kite |  | 1 |  |  |  |
| *Accipiter bicolor* (Vieillot, 1817) | Bicolored Hawk |  | 1 |  |  |  |
| *Rupornis magnirostris* (Gmelin, 1788) | Roadside Hawk |  |  |  | 1 | 1 |
| *Geranoaetus albicaudatus* (Vieillot, 1816) | White-tailed Hawk |  |  |  |  | 1 |
| *Leucopternis kuhli* Bonaparte, 1850 | White-browed Hawk |  | 1 |  |  |  |
| *Buteo nitidus* (Latham, 1790) | Gray Hawk |  |  |  |  | 1 |
| *Buteo brachyurus* Vieillot, 1816 | Short-tailed Hawk |  |  |  | 1 |  |
| Gruiformes Bonaparte, 1854 |  |  |  |  |  |  |
| Rallidae Rafinesque, 1815 |  |  |  |  |  |  |
| *Micropygia schomburgkii* (Cabanis, 1848) | Ocellated Crake |  |  |  |  | 1 |
| *Laterallus viridis* (Statius Muller, 1776) | Russet-crowned Crake |  |  |  | 1 | 1 |
| *Laterallus exilis* (Temminck, 1831) | Gray-breasted Crake |  |  |  |  | 1 |
| *Porzana albicollis* (Vieillot, 1819) | Ash-throated Crake |  |  |  | 1 |  |
| Charadriiformes Huxley, 1867 |  |  |  |  |  |  |
| Charadrii Huxley, 1867 |  |  |  |  |  |  |
| Charadriidae Leach, 1820 |  |  |  |  |  |  |
| *Vanellus chilensis* (Molina, 1782) | Southern Lapwing |  |  |  | 1 |  |
| Scolopaci Steijneger, 1885 |  |  |  |  |  |  |
| Scolopacidae Rafinesque, 1815 |  |  |  |  |  |  |
| *Tringa solitaria* Wilson, 1813 | Solitary Sandpiper |  |  |  | 1 |  |
| Columbiformes Latham, 1790 |  |  |  |  |  |  |
| *Columbina passerina* (Linnaeus, 1758) | Common Ground-Dove |  |  |  | 1 | 1 |
| *Columbina minuta* (Linnaeus, 1766) | Plain-breasted Ground-Dove |  |  |  |  | 1 |
| *Columbina talpacoti* (Temminck, 1811) | Ruddy Ground-Dove |  |  |  | 1 | 1 |
| *Patagioenas speciosa* (Gmelin, 1789) | Scaled Pigeon |  |  | 1 |  |  |
| *Patagioenas picazuro* (Temminck, 1813) | Picazuro Pigeon |  |  |  | 1 |  |
| *Patagioenas plumbea* (Vieillot, 1818) | Plumbeous Pigeon |  | 1 |  |  |  |
| *Patagioenas subvinacea* (Lawrence, 1868) | Ruddy Pigeon | VU | 1 |  |  |  |
| *Leptotila verreauxi* Bonaparte, 1855 | White-tipped Dove |  |  |  | 1 | 1 |
| *Leptotila rufaxilla* (Richard & Bernard, 1792) | Gray-fronted Dove |  |  | 1 |  | 1 |
| Cuculiformes Wagler, 1830 |  |  |  |  |  |  |
| Cuculidae Leach, 1820 |  |  |  |  |  |  |
| Cuculinae Leach, 1820 |  |  |  |  |  |  |
| *Piaya cayana* (Linnaeus, 1766) | Squirrel Cuckoo |  | 1 | 1 | 1 |  |
| Crotophaginae Swainson, 1837 |  |  |  |  |  |  |
| *Crotophaga ani* Linnaeus, 1758 | Smooth-billed Ani |  |  |  | 1 | 1 |
| *Guira guira* (Gmelin, 1788) | Guira Cuckoo |  |  |  |  | 1 |
| Taperinae Verheyen, 1956 |  |  |  |  |  |  |
| *Tapera naevia* (Linnaeus, 1766) | Striped Cuckoo |  |  |  | 1 | 1 |
| *Dromococcyx phasianellus* (Spix, 1824) | Pheasant Cuckoo |  |  |  | 1 |  |
| Strigiformes Wagler, 1830 |  |  |  |  |  |  |
| Strigidae Leach, 1820 |  |  |  |  |  |  |
| *Megascops usta* (Sclater, 1858) | Austral Screech-Owl |  | 1 |  |  |  |
| *Athene cunicularia* (Molina, 1782) | Burrowing Owl |  |  |  | 1 |  |
| Nyctibiiformes Yuri, Kimball, Harshman, Bowie, Braun, Chojnowski, Han, Hackett, Huddleston, Moore, Reddy, Sheldon, Steadman, Witt & Braun, 2013 |  |  |  |  |  |  |
| Nyctibiidae Chenu & Des Murs, 1851 |  |  |  |  |  |  |
| *Nyctibius aethereus* (Wied, 1820) | Long-tailed Potoo |  |  |  |  |  |
| Caprimulgiformes Ridgway, 1881 |  |  |  |  |  |  |
| *Lurocalis semitorquatus* (Gmelin, 1789) | Short-tailed Nighthawk |  | 1 |  |  |  |
| *Hydropsalis nigrescens* (Cabanis, 1848) | Blackish Nightjar |  | 1 |  |  |  |
| *Hydropsalis albicollis* (Gmelin, 1789) | Pauraque |  |  | 1 | 1 |  |
| Apodiformes Peters, 1940 |  |  |  |  |  |  |
| Apodidae Olphe-Galliard, 1887 |  |  |  |  |  |  |
| *Chaetura spinicaudus* (Temminck, 1839) | Band-rumped Swift |  | 1 | 1 | 1 | 1 |
| *Chaetura chapmani* Hellmayr, 1907 | Chapman's Swift |  |  |  | 1 |  |
| *Chaetura brachyura* (Jardine, 1846) | Short-tailed Swift |  | 1 | 1 | 1 | 1 |
| Trochilidae Vigors, 1825 |  |  |  |  |  |  |
| Phaethornithinae Jardine, 1833 |  |  |  |  |  |  |
| *Glaucis hirsutus* (Gmelin, 1788) | Rufous-breasted Hermit |  | 1 | 1 |  | 1 |
| *Phaethornis ruber* (Linnaeus, 1758) | Reddish Hermit |  | 1 | 1 | 1 | 1 |
| *Phaethornis superciliosus* (Linnaeus, 1766) | Long-tailed Hermit |  | 1 |  |  |  |
| Trochilinae Vigors, 1825 |  |  |  |  |  |  |
| *Campylopterus largipennis* (Boddaert, 1783) | Gray-breasted Sabrewing |  | 1 |  |  |  |
| *Anthracothorax nigricollis* (Vieillot, 1817) | Black-throated Mango |  |  |  | 1 | 1 |
| *Thalurania furcata* (Gmelin, 1788) | Fork-tailed Woodnymph |  | 1 |  |  | 1 |
| *Hylocharis cyanus* (Vieillot, 1818) | White-chinned Sapphire |  | 1 |  |  |  |
| *Polytmus theresiae* (Da Silva Maia, 1843) | Green-tailed Goldenthroat |  |  |  | 1 |  |
| Trogoniformes A. O. U., 1886 |  |  |  |  |  |  |
| Trogonidae Lesson, 1828 |  |  |  |  |  |  |
| *Trogon melanurus* Swainson, 1838 | Black-tailed Trogon |  | 1 |  |  |  |
| *Trogon viridis* Linnaeus, 1766 | White-tailed Trogon |  | 1 |  |  |  |
| *Trogon ramonianus* Deville & DesMurs, 1849 | Amazonian Trogon |  | 1 |  |  |  |
| *Trogon rufus* Gmelin, 1788 | Black-throated Trogon |  | 1 |  |  |  |
| Coraciiformes Forbes, 1844 |  |  |  |  |  |  |
| Alcedinidae Rafinesque, 1815 |  |  |  |  |  |  |
| *Megaceryle torquata* (Linnaeus, 1766) | Ringed Kingfisher |  | 1 |  |  |  |
| Momotidae Gray, 1840 |  |  |  |  |  |  |
| *Momotus momota* (Linnaeus, 1766) | Amazonian Motmot |  | 1 | 1 |  |  |
| Galbuliformes Fürbringer, 1888 |  |  |  |  |  |  |
| Galbulidae Vigors, 1825 |  |  |  |  |  |  |
| *Brachygalba lugubris* (Swainson, 1838) | Brown Jacamar |  | 1 |  |  |  |
| *Galbula cyanicollis* Cassin, 1851 | Blue-cheeked Jacamar |  | 1 |  |  |  |
| *Galbula dea* (Linnaeus, 1758) | Paradise Jacamar |  | 1 |  |  |  |
| *Jacamerops aureus* (Statius Muller, 1776) | Great Jacamar |  | 1 |  |  |  |
| Bucconidae Horsfield, 1821 |  |  |  |  |  |  |
| *Notharchus tectus* (Boddaert, 1783) | Pied Puffbird |  | 1 |  |  |  |
| *Bucco capensis* Linnaeus, 1766 | Collared Puffbird |  | 1 |  |  |  |
| *Nystalus striolatus* (Pelzeln, 1856) | Natterer's Striolated Puffbird | **END2** | 1 |  |  |  |
| *Nystalus maculatus* (Gmelin, 1788) | Spot-backed Puffbird |  |  |  | 1 | 1 |
| *Malacoptila rufa* (Spix, 1824) | Rufous-necked Puffbird |  | 1 |  |  |  |
| *Monasa morphoeus* (Hahn & Küster, 1823) | White-fronted Nunbird |  | 1 |  |  |  |
| *Chelidoptera tenebrosa* (Pallas, 1782) | Swallow-winged Puffbird |  |  |  | 1 |  |
| Piciformes Meyer & Wolf, 1810 |  |  |  |  |  |  |
| Ramphastidae Vigors, 1825 |  |  |  |  |  | 1 |
| *Ramphastos tucanus* Linnaeus, 1758 | White-throated Toucan | VU | 19 |  |  |  |
| *Ramphastos vitellinus* Lichtenstein, 1823 | Channel-billed Toucan | EN | 14 | 1 |  |  |
| *Pteroglossus inscriptus* Swainson, 1822 | Lettered Aracari |  |  | 1 |  |  |
| *Pteroglossus bitorquatus* Vigors, 1826 | Red-necked Aracari | EN, **END2** | 3 |  | 1 |  |
| *Pteroglossus aracari* (Linnaeus, 1758) | Black-necked Aracari |  | 9 | 2 |  |  |
| Picidae Leach, 1820 |  |  |  |  |  |  |
| *Melanerpes candidus* (Otto, 1796) | White Woodpecker |  |  |  |  | 1 |
| *Melanerpes cruentatus* (Boddaert, 1783) | Yellow-tufted Woodpecker |  | 1 |  |  |  |
| *Veniliornis affinis* (Swainson, 1821) | Red-stained Woodpecker |  | 1 | 1 |  |  |
| *Piculus flavigula* (Boddaert, 1783) | Yellow-throated Woodpecker |  | 1 |  |  |  |
| *Piculus paraensis* (Snethlage, 1907) | Belem Golden-green Woodpecker | **END1** | 1 |  |  |  |
| *Colaptes melanochloros* (Gmelin, 1788) | Green-barred Woodpecker |  |  |  |  | 1 |
| *Celeus undatus* (Linnaeus, 1766) | Waved Woodpecker |  | 1 |  |  |  |
| *Celeus flavus* (Statius Muller, 1776) | Cream-colored Woodpecker |  | 1 |  | 1 |  |
| *Dryocopus lineatus* (Linnaeus, 1766) | Lineated Woodpecker |  |  |  | 1 |  |
| *Campephilus rubricollis* (Boddaert, 1783) | Red-necked Woodpecker |  | 1 |  | 1 |  |
| Falconiformes Bonaparte, 1831 |  |  |  |  |  |  |
| Falconidae Leach, 1820 |  |  |  |  |  |  |
| *Ibycter americanus* (Boddaert, 1783) | Red-throated Caracara |  | 1 |  |  |  |
| *Caracara plancus* (Miller, 1777) | Southern Caracara |  |  |  | 1 | 1 |
| *Milvago chimachima* (Vieillot, 1816) | Yellow-headed Caracara |  |  |  | 1 |  |
| *Micrastur ruficollis* (Vieillot, 1817) | Barred Forest-Falcon |  | 1 |  |  |  |
| *Micrastur mintoni* Whittaker, 2002 | Cryptic Forest-Falcon | **END2** | 1 |  |  |  |
| Psittaciformes Wagler, 1830 |  |  |  |  |  |  |
| Psittacidae Rafinesque, 1815 |  |  |  |  |  |  |
| *Ara macao* (Linnaeus, 1758) | Scarlet Macaw |  | 1 |  |  |  |
| *Guaruba guarouba* (Gmelin, 1788) | Golden Parakeet | VU, **END2** | 1 |  | 1 |  |
| *Pyrrhura lepida* (Wagler, 1832) | Pearly Parakeet | VU, **END2** | 1 |  |  |  |
| *Brotogeris chrysoptera* (Linnaeus, 1766) | Golden-winged Parakeet |  | 1 |  | 1 |  |
| *Pionites leucogaster* (Kuhl, 1820) | White-bellied Parrot | VU | 1 |  |  |  |
| *Pyrilia vulturina* (Kuhl, 1820) | Vulturine Parrot | VU, **END2** | 1 |  |  |  |
| *Pionus menstruus* (Linnaeus, 1766) | Blue-headed Parrot |  | 1 | 1 | 1 | 1 |
| *Pionus fuscus* (Statius Muller, 1776) | Dusky Parrot |  | 1 |  |  | 1 |
| *Amazona farinosa* (Boddaert, 1783) | Mealy Parrot |  | 1 |  |  |  |
| *Amazona amazonica* (Linnaeus, 1766) | Orange-winged Parrot |  | 1 | 1 | 1 | 1 |
| *Deroptyus accipitrinus* (Linnaeus, 1758) | Red-fan Parrot |  | 1 | 1 |  |  |
| Passeriformes Linnaeus, 1758 |  |  |  |  |  |  |
| Tyranni Wetmore & Miller, 1926 |  |  |  |  |  |  |
| Thamnophilida Patterson, 1987 |  |  |  |  |  |  |
| Thamnophilidae Swainson, 1824 |  |  |  |  |  |  |
| Myrmornithinae Sundevall, 1872 |  |  |  |  |  |  |
| *Pygiptila stellaris* (Spix, 1825) | Spot-winged Antshrike |  | 1 |  |  |  |
| Thamnophilinae Swainson, 1824 |  |  |  |  |  |  |
| *Myrmotherula axillaris* (Vieillot, 1817) | White-flanked Antwren |  | 1 | 1 |  |  |
| *Myrmotherula longipennis* Pelzeln, 1868 | Long-winged Antwren |  | 1 |  |  |  |
| *Myrmotherula menetriesii* (d'Orbigny, 1837) | Gray Antwren |  | 1 |  |  |  |
| *Formicivora grisea* (Boddaert, 1783) | White-fringed Antwren |  |  | 1 | 1 | 1 |
| *Formicivora rufa* (Wied, 1831) | Rusty-backed Antwren |  |  |  |  | 1 |
| *Isleria hauxwelli* (Sclater, 1857) | Plain-throated Antwren |  | 1 |  |  |  |
| *Thamnomanes caesius* (Temminck, 1820) | Cinereous Antshrike |  | 1 |  |  |  |
| *Dysithamnus mentalis* (Temminck, 1823) | Plain Antvireo |  | 1 |  |  |  |
| *Herpsilochmus rufimarginatus* (Temminck, 1822) | Rufous-winged Antwren |  | 1 |  |  |  |
| *Thamnophilus palliatus* (Lichtenstein, 1823) | Chestnut-backed Antshrike |  |  | 1 | 1 |  |
| *Thamnophilus aethiops* Sclater, 1858 | White-shouldered Antshrike | **END1*** | 1 |  |  |  |
| *Thamnophilus amazonicus* Sclater, 1858 | Amazonian Antshrike |  | 1 | 1 | 1 |  |
| *Taraba major* (Vieillot, 1816) | Great Antshrike |  | 1 | 1 | 1 | 1 |
| *Pyriglena leuconota* (Spix, 1824) | White-backed Fire-eye |  | 1 | 1 |  |  |
| *Cercomacra cinerascens* (Sclater, 1857) | Gray Antbird |  | 1 |  |  |  |
| *Cercomacra laeta* Todd, 1920 | Willis's Antbird |  | 1 | 1 |  |  |
| *Willisornis vidua* (Hellmayr, 1905) | Xingu Scale-backed Antbird | **END2** | 1 | 1 |  |  |
| *Phlegopsis nigromaculata* (d'Orbigny & Lafresnaye, 1837) | Black-spotted Bare-eye | **END1*** | 1 |  |  |  |
| Conopophagidae Sclater & Salvin, 1873 |  |  |  |  |  |  |
| *Conopophaga roberti* Hellmayr, 1905 | Hooded Gnateater | **END1** | 1 | 1 |  |  |
| Furnariida Sibley, Ahlquist & Monroe, 1988 |  |  |  |  |  |  |
| Furnarioidea Gray, 1840 |  |  |  |  |  |  |
| Formicariidae Gray, 1840 |  |  |  |  |  |  |
| *Formicarius analis* (d'Orbigny & Lafresnaye, 1837) | Black-faced Antthrush |  | 1 |  |  |  |
| Dendrocolaptidae Gray, 1840 |  |  |  |  |  |  |
| Sittasominae Ridgway, 1911 |  |  |  |  |  |  |
| *Dendrocincla fuliginosa* (Vieillot, 1818) | Plain-brown Woodcreeper |  | 1 |  |  |  |
| *Deconychura longicauda* (Pelzeln, 1868) | Long-tailed Woodcreeper | VU | 1 |  |  |  |
| *Certhiasomus stictolaemus* (Pelzeln, 1868) | Spot-throated Woodcreeper |  | 1 |  |  |  |
| Dendrocolaptinae Gray, 1840 |  |  |  |  |  |  |
| *Glyphorynchus spirurus* (Vieillot, 1819) | Wedge-billed Woodcreeper |  | 1 | 1 |  |  |
| *Xiphorhynchus spixii* (Lesson, 1830) | Spix's Woodcreeper | **END2** | 1 |  |  |  |
| *Xiphorhynchus guttatus* (Lichtenstein, 1820) | Buff-throated Woodcreeper |  | 1 |  |  |  |
| *Dendroplex picus* (Gmelin, 1788) | Straight-billed Woodcreeper |  |  | 1 | 1 |  |
| *Lepidocolaptes layardi* (Sclater, 1873) | Layard's Woodcreeper | **END2** | 1 |  |  |  |
| *Dendrocolaptes medius* Todd, 1920 | Todd's Woodcreeper | **END1** | 1 |  |  |  |
| Xenopidae Bonaparte, 1854 |  |  |  |  |  |  |
| *Xenops minutus* (Sparrman, 1788) | Plain Xenops |  | 1 |  |  |  |
| Furnariidae Gray, 1840 |  |  |  |  |  |  |
| Philydorinae Sclater & Salvin, 1873 |  |  |  |  |  |  |
| *Automolus paraensis* Hartert, 1902 | Para Foliage-gleaner | **END2** | 1 |  |  |  |
| *Anabacerthia ruficaudata* (d'Orbigny & Lafresnaye, 1838) | Rufous-tailed Foliage-gleaner |  | 1 |  |  |  |
| *Philydor erythrocercum* (Pelzeln, 1859) | Rufous-rumped Foliage-gleaner |  | 1 |  |  |  |
| *Synallaxis albescens* Temminck, 1823 | Pale-breasted Spinetail |  |  |  | 1 | 1 |
| *Synallaxis rutilans* Temminck, 1823 | Ruddy Spinetail | **END1*** | 1 |  |  |  |
| Tyrannida Wetmore & Miller, 1926 |  |  |  |  |  |  |
| Pipridae Rafinesque, 1815 |  |  |  |  |  |  |
| Neopelminae Tello, Moyle, Marchese & Cracraft, 2009 |  |  |  |  |  |  |
| *Tyranneutes stolzmanni* (Hellmayr, 1906) | Dwarf Tyrant-Manakin |  |  |  |  |  |
| Piprinae Rafinesque, 1815 |  |  |  |  |  |  |
| *Ceratopipra rubrocapilla* (Temminck, 1821) | Red-headed Manakin |  | 1 |  |  |  |
| *Manacus manacus* (Linnaeus, 1766) | White-bearded Manakin |  | 1 | 1 | 1 |  |
| *Dixiphia pipra* (Linnaeus, 1758) | White-crowned Manakin |  | 1 |  |  |  |
| Ilicurinae Prum, 1992 |  |  |  |  |  |  |
| *Chiroxiphia pareola* (Linnaeus, 1766) | Blue-backed Manakin |  | 1 | 1 |  |  |
| Cotingoidea Bonaparte, 1849 |  |  |  |  |  |  |
| *Onychorhynchus coronatus* (Statius Muller, 1776) | Royal Flycatcher |  | 1 |  |  |  |
| *Terenotriccus erythrurus* (Cabanis, 1847) | Ruddy-tailed Flycatcher |  | 1 |  |  |  |
| *Myiobius barbatus* (Gmelin, 1789) | Whiskered Flycatcher |  | 1 |  |  |  |
| Tityridae Gray, 1840 |  |  |  |  |  |  |
| Schiffornithinae Sibley & Ahlquist, 1985 |  |  |  |  |  |  |
| *Schiffornis turdina* (Wied, 1831) | Thrush-like Schiffornis |  | 1 |  |  |  |
| Tityrinae Gray, 1840 |  |  |  |  |  |  |
| *Tityra cayana* (Linnaeus, 1766) | Black-tailed Tityra |  | 1 |  |  |  |
| *Pachyramphus rufus* (Boddaert, 1783) | Cinereous Becard |  |  | 1 | 1 |  |
| *Pachyramphus marginatus* (Lichtenstein, 1823) | Black-capped Becard |  | 1 |  |  |  |
| *Pachyramphus minor* (Lesson, 1830) | Pink-throated Becard |  | 1 |  |  |  |
| Cotingidae Bonaparte, 1849 |  |  |  |  |  |  |
| Cotinginae Bonaparte, 1849 |  |  |  |  |  |  |
| *Lipaugus vociferans* (Wied, 1820) | Screaming Piha |  | 1 |  |  |  |
| *Cotinga cayana* (Linnaeus, 1766) | Spangled Cotinga |  | 1 |  |  |  |
| *Querula purpurata* (Statius Muller, 1776) | Purple-throated Fruitcrow |  | 1 |  |  |  |
| Tyrannoidea Vigors, 1825 |  |  |  |  |  |  |
| Pipritidae Ohlson, Irestedt, Ericson & Fjeldså, 2013 |  |  |  |  |  |  |
| *Piprites chloris* (Temminck, 1822) | Wing-barred Piprites | **END1*** | 1 |  |  |  |
| Platyrinchidae Bonaparte, 1854 |  |  |  |  |  |  |
| *Platyrinchus saturatus* Salvin & Godman, 1882 | Cinnamon-crested Spadebill |  | 1 |  |  |  |
| *Platyrinchus platyrhynchos* (Gmelin, 1788) | White-crested Spadebill |  | 1 |  |  |  |
| Rhynchocyclidae Berlepsch, 1907 |  |  | 1 |  |  |  |
| *Incertae sedis* |  |  |  |  |  |  |
| *Taeniotriccus andrei* (Berlepsch & Hartert, 1902) | Black-chested Tyrant |  | 1 | 1 |  |  |
| Pipromorphinae Wolters, 1977 |  |  | 1 |  |  |  |
| *Mionectes macconnelli* (Chubb, 1919) | McConnell's Flycatcher |  | 1 |  |  |  |
| Rhynchocyclinae Berlepsch, 1907 |  |  |  |  |  |  |
| *Rhynchocyclus olivaceus* (Temminck, 1820) | Olivaceous Flatbill |  | 1 |  |  |  |
| *Tolmomyias sulphurescens* (Spix, 1825) | Yellow-olive Flycatcher |  | 1 |  |  |  |
| *Tolmomyias assimilis* (Pelzeln, 1868) | Yellow-margined Flycatcher |  | 1 |  |  |  |
| *Tolmomyias poliocephalus* (Taczanowski, 1884) | Gray-crowned Flycatcher |  |  | 1 |  |  |
| *Tolmomyias flaviventris* (Wied, 1831) | Yellow-breasted Flycatcher |  | 1 | 1 | 1 | 1 |
| *Todirostrum cinereum* (Linnaeus, 1766) | Common Tody-Flycatcher |  |  |  | 1 |  |
| *Todirostrum chrysocrotaphum* Strickland, 1850 | Yellow-browed Tody-Flycatcher |  | 1 |  |  |  |
| *Poecilotriccus fumifrons* (Hartlaub, 1853) | Smoky-fronted Tody-Flycatcher |  |  |  | 1 |  |
| *Poecilotriccus sylvia* (Desmarest, 1806) | Slate-headed Tody-Flycatcher |  |  | 1 | 1 |  |
| *Myiornis sp. novum* | undescribed pygmy-tyrant | **END1** | 1 |  |  |  |
| *Myiornis ecaudatus* (d'Orbigny & Lafresnaye, 1837) | Short-tailed Pygmy-Tyrant |  | 1 |  |  |  |
| *Lophotriccus galeatus* (Boddaert, 1783) | Helmeted Pygmy-Tyrant |  | 1 | 1 |  |  |
| Tyrannidae Vigors, 1825 |  |  |  |  |  |  |
| Elaeniinae Cabanis & Heine, 1860 |  |  |  |  |  |  |
| *Zimmerius gracilipes* (Sclater & Salvin, 1868) | Slender-footed Tyrannulet |  | 1 | 1 |  |  |
| *Ornithion inerme* Hartlaub, 1853 | White-lored Tyrannulet |  | 1 | 1 |  |  |
| *Camptostoma obsoletum* (Temminck, 1824) | Southern Beardless-Tyrannulet |  | 1 | 1 | 1 | 1 |
| *Elaenia flavogaster* (Thunberg, 1822) | Yellow-bellied Elaenia |  | 1 | 1 | 1 | 1 |
| *Elaenia chiriquensis* Lawrence, 1865 | Lesser Elaenia |  |  |  |  | 1 |
| *Myiopagis gaimardii* (d'Orbigny, 1839) | Forest Elaenia |  | 1 | 1 |  |  |
| *Myiopagis caniceps* (Swainson, 1835) | Gray Elaenia |  | 1 |  |  |  |
| *Tyrannulus elatus* (Latham, 1790) | Yellow-crowned Tyrannulet |  | 1 | 1 | 1 |  |
| *Phaeomyias murina* (Spix, 1825) | Mouse-colored Tyrannulet |  |  | 1 | 1 | 1 |
| Tyranninae Vigors, 1825 |  |  |  |  |  |  |
| *Attila spadiceus* (Gmelin, 1789) | Bright-rumped Attila |  | 1 |  |  |  |
| *Legatus leucophaius* (Vieillot, 1818) | Piratic Flycatcher |  |  | 1 | 1 |  |
| *Myiarchus tuberculifer* (d'Orbigny & Lafresnaye, 1837) | Dusky-capped Flycatcher |  | 1 |  |  |  |
| *Myiarchus ferox* (Gmelin, 1789) | Short-crested Flycatcher |  |  | 1 | 1 | 1 |
| *Myiarchus tyrannulus* (Statius Muller, 1776) | Brown-crested Flycatcher |  | 1 | 1 | 1 | 1 |
| *Rhytipterna simplex* (Lichtenstein, 1823) | Grayish Mourner |  | 1 |  |  |  |
| *Casiornis fuscus* Sclater & Salvin, 1873 | Ash-throated Casiornis |  |  | 1 |  |  |
| *Pitangus sulphuratus* (Linnaeus, 1766) | Great Kiskadee |  | 1 | 1 | 1 | 1 |
| *Myiodynastes maculatus* (Statius Muller, 1776) | Streaked Flycatcher |  |  |  | 1 |  |
| *Megarynchus pitangua* (Linnaeus, 1766) | Boat-billed Flycatcher |  | 1 |  | 1 |  |
| *Myiozetetes cayanensis* (Linnaeus, 1766) | Rusty-margined Flycatcher |  | 1 |  | 1 |  |
| *Tyrannus melancholicus* Vieillot, 1819 | Tropical Kingbird |  |  |  | 1 | 1 |
| *Empidonomus varius* (Vieillot, 1818) | Variegated Flycatcher |  |  |  | 1 |  |
| Fluvicolinae Swainson, 1832 |  |  |  |  |  |  |
| *Colonia colonus* (Vieillot, 1818) | Long-tailed Tyrant |  | 1 |  |  |  |
| *Myiophobus fasciatus* (Statius Muller, 1776) | Bran-colored Flycatcher |  |  | 1 | 1 | 1 |
| *Sublegatus obscurior* Todd, 1920 | Amazonian Scrub-Flycatcher |  | 1 |  |  |  |
| *Arundinicola leucocephala* (Linnaeus, 1764) | White-headed Marsh Tyrant |  |  |  | 1 |  |
| Passeri Linnaeus, 1758 |  |  |  |  |  |  |
| Corvida Wagler 1830 |  |  |  |  |  |  |
| Vireonidae Swainson, 1837 |  |  |  |  |  |  |
| *Cyclarhis gujanensis* (Gmelin, 1789) | Rufous-browed Peppershrike |  | 1 |  | 1 |  |
| *Hylophilus semicinereus* Sclater & Salvin, 1867 | Gray-chested Greenlet |  | 1 | 1 |  | 1 |
| *Hylophilus pectoralis* Sclater, 1866 | Ashy-headed Greenlet |  |  |  | 1 |  |
| Passerida Linnaeus, 1758 |  |  |  |  |  |  |
| Hirundinidae Rafinesque, 1815 |  |  |  |  |  |  |
| *Stelgidopteryx ruficollis* (Vieillot, 1817) | Southern Rough-winged Swallow |  |  |  | 1 | 1 |
| *Progne tapera* (Vieillot, 1817) | Brown-chested Martin |  |  |  | 1 | 1 |
| *Progne chalybea* (Gmelin, 1789) | Gray-breasted Martin |  | 1 | 1 | 1 | 1 |
| *Tachycineta albiventer* (Boddaert, 1783) | White-winged Swallow |  |  |  | 1 |  |
| *Hirundo rustica* Linnaeus, 1758 | Barn Swallow |  |  |  | 1 | 1 |
| Troglodytidae Swainson, 1831 |  |  |  |  |  |  |
| *Microcerculus marginatus* (Sclater, 1855) | Scaly-breasted Wren |  | 1 |  |  |  |
| *Troglodytes musculus* Naumann, 1823 | Southern House Wren |  |  | 1 | 1 | 1 |
| *Pheugopedius genibarbis* (Swainson, 1838) | Moustached Wren |  | 1 | 1 | 1 |  |
| Polioptilidae Baird, 1858 |  |  |  |  |  |  |
| *Ramphocaenus melanurus* Vieillot, 1819 | Long-billed Gnatwren |  | 1 | 1 |  |  |
| *Polioptila plumbea* (Gmelin, 1788) | Tropical Gnatcatcher |  | 1 | 1 | 1 |  |
| Turdidae Rafinesque, 1815 |  |  |  |  |  |  |
| *Turdus leucomelas* Vieillot, 1818 | Pale-breasted Thrush |  | 1 | 1 | 1 | 1 |
| Motacillidae Horsfield, 1821 |  |  |  |  |  |  |
| *Anthus lutescens* Pucheran, 1855 | Yellowish Pipit |  |  |  | 1 | 1 |
| Passerellidae Cabanis & Heine, 1850 |  |  |  |  |  | 1 |
| *Ammodramus humeralis* (Bosc, 1792) | Grassland Sparrow |  |  | 1 | 1 | 1 |
| *Ammodramus aurifrons* (Spix, 1825) | Yellow-browed Sparrow |  |  |  | 1 | 1 |
| *Arremon taciturnus* (Hermann, 1783) | Pectoral Sparrow |  | 1 |  |  |  |
| Parulidae Wetmore, Friedmann, Lincoln, Miller, Peters, van Rossem, Van Tyne & Zimmer 1947 |  |  |  |  |  |  |
| *Geothlypis aequinoctialis* (Gmelin, 1789) | Masked Yellowthroat |  |  |  | 1 | 1 |
| Icteridae Vigors, 1825 |  |  |  |  |  |  |
| *Psarocolius viridis* (Statius Muller, 1776) | Green Oropendola |  | 1 |  |  | 1 |
| *Psarocolius bifasciatus* (Spix, 1824) | Olive Oropendola |  | 1 |  |  |  |
| *Cacicus cela* (Linnaeus, 1758) | Yellow-rumped Cacique |  | 1 |  | 1 | 1 |
| *Molothrus bonariensis* (Gmelin, 1789) | Shiny Cowbird |  |  |  | 1 |  |
| *Sturnella militaris* (Linnaeus, 1758) | Red-breasted Blackbird |  |  | 1 | 1 | 1 |
| Mitrospingidae Barker, Burns, Klicka, Lanyon & Lovette, 2013 |  |  |  |  |  |  |
| *Lamprospiza melanoleuca* (Vieillot, 1817) | Red-billed Pied Tanager |  | 1 |  |  |  |
| Thraupidae Cabanis, 1847 |  |  |  |  |  |  |
| *Coereba flaveola* (Linnaeus, 1758) | Bananaquit |  | 1 | 1 | 1 | 1 |
| *Saltator maximus* (Statius Muller, 1776) | Buff-throated Saltator |  | 1 | 1 | 1 |  |
| *Saltator azarae* d’Orbigny, 1839 | Amazonian Grayish Saltator |  |  | 1 | 1 |  |
| *Saltator grossus* (Linnaeus, 1766) | Slate-colored Grosbeak |  | 1 |  |  |  |
| *Tachyphonus rufus* (Boddaert, 1783) | White-lined Tanager |  |  | 1 | 1 | 1 |
| *Ramphocelus carbo* (Pallas, 1764) | Silver-beaked Tanager |  | 1 | 1 | 1 | 1 |
| *Lanio luctuosus* (d'Orbigny & Lafresnaye, 1837) | White-shouldered Tanager | 1 | 1 |  |  |  |
| *Lanio cristatus* (Linnaeus, 1766) | Flame-crested Tanager | 1 | 1 |  |  |  |
| *Lanio surinamus* (Linnaeus, 1766) | Fulvous-crested Tanager | 1 | 1 |  |  |  |
| *Tangara gyrola* (Linnaeus, 1758) | Bay-headed Tanager |  | 1 |  |  |  |
| *Tangara mexicana* (Linnaeus, 1766) | Turquoise Tanager |  | 1 |  |  |  |
| *Tangara punctata* (Linnaeus, 1766) | Spotted Tanager |  | 1 |  |  |  |
| *Tangara episcopus* (Linnaeus, 1766) | Blue-gray Tanager |  | 1 | 1 | 1 | 1 |
| *Tangara palmarum* (Wied, 1823) | Palm Tanager |  | 1 | 1 | 1 | 1 |
| *Schistochlamys melanopis* (Latham, 1790) | Black-faced Tanager |  |  |  | 1 | 1 |
| *Cyanerpes caeruleus* (Linnaeus, 1758) | Purple Honeycreeper |  |  |  | 1 |  |
| *Emberizoides herbicola* (Vieillot, 1817) | Wedge-tailed Grass-Finch |  |  |  | 1 | 1 |
| *Volatinia jacarina* (Linnaeus, 1766) | Blue-black Grassquit |  |  |  | 1 | 1 |
| *Sporophila plumbea* (Wied, 1830) | Plumbeous Seedeater |  |  |  | 1 | 1 |
| *Sporophila americana* (Gmelin, 1789) | Wing-barred Seedeater |  |  |  | 1 |  |
| *Sporophila nigricollis* (Vieillot, 1823) | Yellow-bellied Seedeater |  |  |  | 1 | 1 |
| *Sporophila minuta* (Linnaeus, 1758) | Ruddy-breasted Seedeater |  |  |  | 1 |  |
| *Sporophila angolensis* (Linnaeus, 1766) | Chestnut-bellied Seed-Finch |  |  | 1 | 1 | 1 |
| Cardinalidae Ridgway, 1901 |  |  |  |  |  |  |
| *Granatellus pelzelni* Sclater, 1865 | Rose-breasted Chat |  | 1 |  |  |  |
| *Caryothraustes canadensis* (Linnaeus, 1766) | Yellow-green Grosbeak |  | 1 | 1 |  |  |
| *Cyanoloxia rothschildii* (Bartlett, 1890) | Rothschild's Blue Grosbeak |  | 1 |  |  |  |
| Fringillidae Leach, 1820 |  |  |  |  |  |  |
| *Euphonia minuta* Cabanis, 1849 | White-vented Euphonia |  | 1 |  |  |  |
| *Euphonia cayennensis* (Gmelin, 1789) | Golden-sided Euphonia |  | 1 |  |  |  |

**Table B.** Top ranked model results from GLMs for the whole avian community and forest birds alone. The explanatory variables include distance to the forest border (Border), tree species richness (Tree richness), forest cover (% of primary forest cover) and biomass of trees (Biomass). For each model R² is the proportion of variation explained, ΔAICc is the difference between AICc between this and the preceding model and weight is the Akaike weight for the given model.

| All birds | | | | |
| --- | --- | --- | --- | --- |
| Model rank | R² | AICc | ΔAICc | Weight |
| Border + Tree richness | 0.64 | 358.49 | 0.00 | 0.26 |
| Border + Cover + Tree richness | 0.66 | 358.79 | 0.30 | 0.22 |
| Tree richness | 0.62 | 359.33 | 0.84 | 0.17 |
| Biomass + Border + Tree richness | 0.66 | 361.28 | 2.80 | 0.06 |
| Cover + Tree richness | 0.62 | 361.46 | 2.97 | 0.06 |
| Forest Birds | | | | |
| Model rank | R² | AICc | ΔAICc | Weight |
| Tree richness | 0.81 | 343.04 | 0.00 | 0.38 |
| Biomass + Tree richness | 0.81 | 344.84 | 1.80 | 0.15 |
| Border + Tree richness | 0.81 | 345.04 | 2.00 | 0.14 |
| Cover + Tree richness | 0.81 | 345.20 | 2.16 | 0.13 |
| Biomass + Cover + Tree richness | 0.81 | 346.68 | 3.64 | 0.06 |

**Table C.** PERMANOVA Pseudo-F statistic values of the global test and P-value and t values of pair-wise comparison, P-values and mean similarity of bird community composition in different land-use types.

|  |  |  |  |
| --- | --- | --- | --- |
| **Groups** | **F-value** | **P-value** | **mean similarity** |
| Global test Land use groups | 8.172 | 0.001 | n/a |
| Oil Palm, Primary Forest | 4.464 | 0.001 | 6.18 |
| Oil Palm, Pasture | 1.765 | 0.005 | 30.46 |
| Oil Palm, Secondary Forest | 2.051 | 0.002 | 21.99 |
| Primary Forest, Pasture | 4.4 | 0.001 | 11.06 |
| Primary Forest, Secondary Forest | 2.378 | 0.001 | 29.44 |
| Pasture, Secondary Forest | 2.131 | 0.001 | 26.05 |

**Figure A.** Species rarefaction curves per point count considering the entire avian assemblage in land-uses (A) primary forest, (B) secondary forest, (C) cattle pasture & (D) oil palm.

**Figure B.** Relationship between distance to the nearest primary forest border and richness of forest bird species for nom primary forest transects around Moju (heavy dark border) and Paragominas (narrow dark border). Green circles denote secondary forest transects, orange circles = cattle pasture, grey circles = mechanised agriculture and red circles = oil palm. Oil palm transects have a comparable species richness to other non-forest land-uses.

**
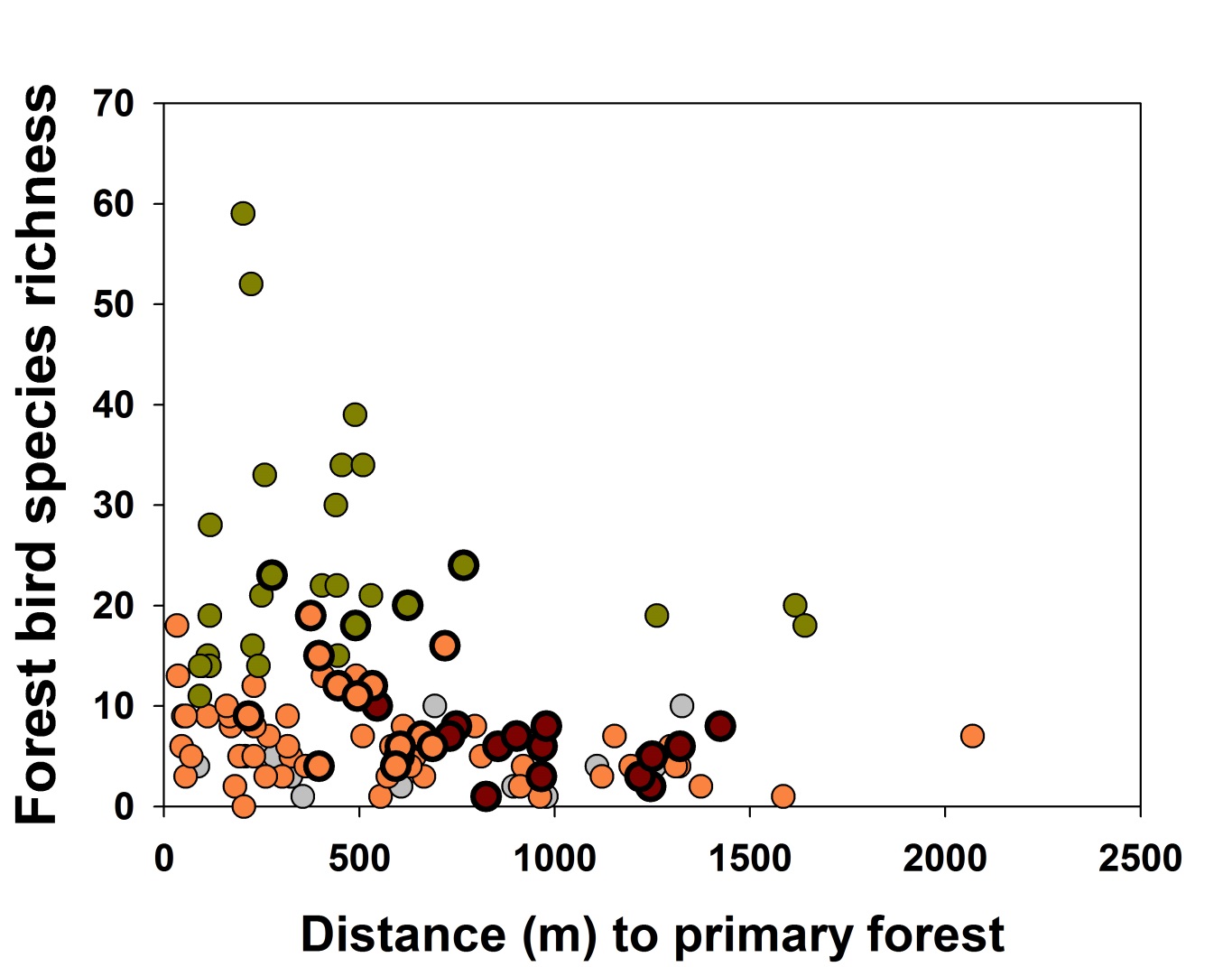
**

**Figure C.** nMDS plot of community structure of the entire avian assemblage in Moju, primary forest transects are represented by dark green squares, secondary forests by light green squares, cattle pastures are yellow circles, the blue star is a natural *campina* formation, dark red triangles are older oil palm plantations (12-25 years), lighter red triangles are intermediate aged oil palm plantations (3-4 years) and orange triangles are recently-planted oil palm plantations (1-2 years). Polygon size is proportional to species richness.
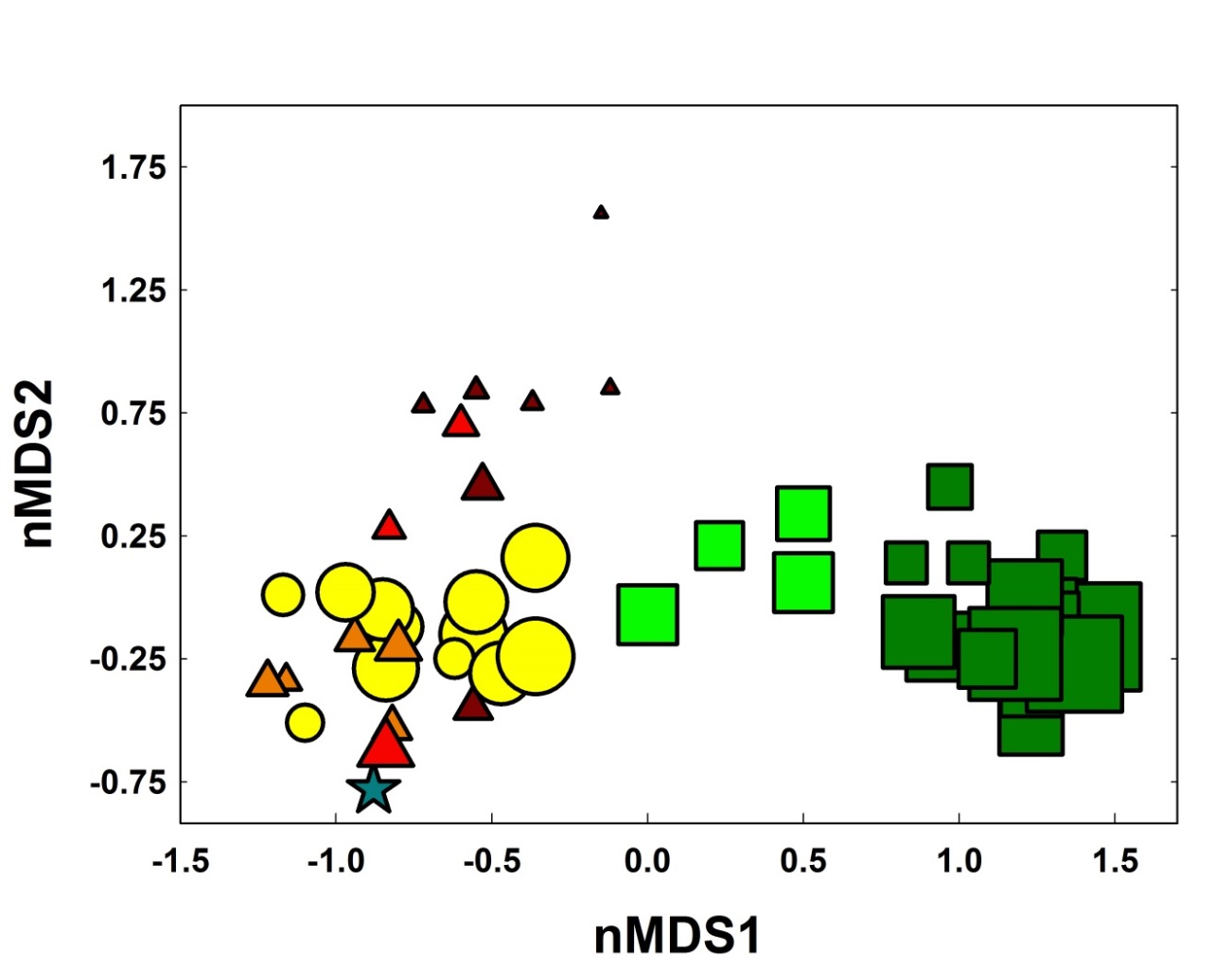

Supplement: S1 File — Systematic list (following CBRO 2014) of bird species recorded in the land-uses: PF = primary forest, SF = secondary forest, CP = cattle pasture, OP = oil palm. The status column highlights both their global Red List status following Birdlife International (2014), where VU = Vulnerable and EN = Endangered and their endemicity (following HBW 2015), where END1 = full species endemic to the Belém AE (and adjacent forests of a similar physiognomy in north-east Brazil), END1* = subspecies endemic to the Belém AE and END2 = species endemic to south-east Amazonia, west of the river Madeira and south of the river Amazon. Table B. Top ranked model results from GLMs for the whole avian community and forest birds alone. The explanatory variables include distance to the forest border (Border), tree species richness (Tree richness), forest cover (% of primary forest cover) and biomass of trees (Biomass). For each model R2 is the proportion of variation explained, ΔAICc is the difference between AICc between this and the preceding model and weight is the Akaike weight for the given model. Table C. PERMANOVA Pseudo-F statistic values of the global test and P-value and t values of pair-wise comparison, P-values and mean similarity of bird community composition in different land-use types. Fig. A. Species rarefaction curves per point count considering the entire avian assemblage in land-uses (A) primary forest, (B) secondary forest, (C) cattle pasture & (D) oil palm. Fig. B. Relationship between distance to the nearest primary forest border and richness of forest bird species for nom primary forest transects around Moju (heavy dark border) and Paragominas (narrow dark border). Green circles denote secondary forest transects, orange circles = cattle pasture, grey circles = mechanised agriculture and red circles = oil palm. Oil palm transects have a comparable species richness to other non-forest land-uses. Fig. C. nMDS plot of community structure of the entire avian assemblage in Moj [file pone.0122432.s001.docx]
